# A Neuron-Specific Antiviral Mechanism Prevents Lethal Flaviviral Infection of Mosquitoes

**DOI:** 10.1371/journal.ppat.1004848

**Published:** 2015-04-27

**Authors:** Xiaoping Xiao, Rudian Zhang, Xiaojing Pang, Guodong Liang, Penghua Wang, Gong Cheng

**Affiliations:** 1 Department of Basic Medical Sciences, School of Medicine, Tsinghua University, Beijing, People's Republic of China; 2 Collaborative Innovation Center for Diagnosis and Treatment of Infectious Diseases, Hangzhou, People's Republic of China; 3 School of Life Science, Tsinghua University, Beijing, People's Republic of China; 4 State Key Laboratory for Infectious Disease Prevention and Control, National Institute for Viral Disease Control and Prevention, Chinese Center for Viral Disease Control and Prevention, Beijing, People's Republic of China; 5 Department of Microbiology and Immunology, School of Medicine, New York Medical College, Valhalla, New York, United States of America; University of Massachusetts Medical School, UNITED STATES

## Abstract

Mosquitoes are natural vectors for many etiologic agents of human viral diseases. Mosquito-borne flaviviruses can persistently infect the mosquito central nervous system without causing dramatic pathology or influencing the mosquito behavior and lifespan. The mechanism by which the mosquito nervous system resists flaviviral infection is still largely unknown. Here we report that an *Aedes aegypti* homologue of the neural factor *Hikaru genki* (*AaHig*) efficiently restricts flavivirus infection of the central nervous system. *AaHig* was predominantly expressed in the mosquito nervous system and localized to the plasma membrane of neural cells. Functional blockade of AaHig enhanced Dengue virus (DENV) and Japanese encephalitis virus (JEV), but not Sindbis virus (SINV), replication in mosquito heads and consequently caused neural apoptosis and a dramatic reduction in the mosquito lifespan. Consistently, delivery of recombinant AaHig to mosquitoes reduced viral infection. Furthermore, the membrane-localized AaHig directly interfaced with a highly conserved motif in the surface envelope proteins of DENV and JEV, and consequently interrupted endocytic viral entry into mosquito cells. Loss of either plasma membrane targeting or virion-binding ability rendered AaHig nonfunctional. Interestingly, *Culex pipien pallens* Hig also demonstrated a prominent anti-flavivirus activity, suggesting a functionally conserved function for Hig. Our results demonstrate that an evolutionarily conserved antiviral mechanism prevents lethal flaviviral infection of the central nervous system in mosquitoes, and thus may facilitate flaviviral transmission in nature.

## Author Summary

The central nervous system plays a predominant role in organisms associated with cognition and higher-order functions, which is key to their normal behavior and successful survival. Many mosquito-borne flaviviruses particularly invade the central nervous system in vertebrates, resulting in dramatic neural degeneration and damage. As natural vectors, mosquitoes are highly permissive to flaviviral infection that can be persistent in the mosquito nervous system. However, the infection intriguingly does neither lead to significant malignant pathological sequelae, nor dramatically influences mosquito behavior or lifespan, and thus mosquitoes can transmit viruses efficiently. Little is known about the neuron-specific resistant mechanism in viral infection of mosquitoes. Here we report that a neuron-specific factor specifically controls flaviviral replication in the mosquito nervous system by interfering with viral entry, and its activity prevents lethal flaviviral infection of mosquitoes. Our study provides insight into the sophisticated interactions between mosquito-borne viruses and their vectors, and offers an important target for arboviral limitation in nature.

## Introduction

Mosquitoes transmit many human pathogens of medical importance throughout the world. Flaviviruses, such as West Nile (WNV), Japanese Encephalitis (JEV), Dengue (DENV) and Yellow Fever (YFV) viruses that are transmitted by mosquitoes are the etiologic agents of human hemorrhagic fever, encephalitis and meningitis [1]. As natural vectors, mosquitoes are very permissive to and allow systematic and persistent flavivirus infection [2,3]. For example, WNV infection is persistent in many tissues of mosquitoes, including the nervous system, salivary glands, midgut, and fat body [4]. The head of mosquitoes, where the central neural system locates, can maintain productive flavivirus infection [4]. Unlike human infection, which can cause severe neurological sequelae, flaviviral infection of the mosquito nervous system intriguingly does not lead to significant malignant pathological consequences, and also does not dramatically influence mosquito behavior or lifespan [5,6,7,8]. The ability of the neural antiviral mechanisms to control viral replication and to maintain a normal mosquito lifespan may facilitate viral dissemination in nature. However, the machinery that controls flavivirus infection of the mosquito nervous system is still largely unknown.

*Hikaru genki* (*Hig*) is predominantly expressed in the pupal and adult nervous system of *Drosophila* and is crucial for the development of neural circuits [9,10]. The *Hig* gene encodes multiple immune-related domains, including an immunoglobulin (Ig) domain and five complement control protein (CCP) domains (also designated Sushi repeat domains) [9]. The Hig protein is therefore speculated to be an immune factor in *Drosophila*, in addition to its role in neural development [9]. The CCP domain is a signature module present in many mammalian complement proteins and is shown to mediate protein-protein interactions of complement components or to recognize microbial pathogens [11]. Human complement receptor 2, encoding 16 CCPs, serves as a cellular entry receptor for Epstein-Barr virus, and its CCP-1 and -2 domains are required for Epstein-Barr virus binding [12]. The membrane cofactor protein (MCP), with 4 CCP repeats, has been demonstrated to function as a cellular receptor for Measles virus [13]. Another complement regulator, Factor H, encoding 20 CCP repeats in the protein, was reported to bind the human immunodeficiency virus (HIV) surface glycoproteins gp41 and gp120 [14,15]. Our previous study also identified that an insect-specific scavenger receptor (SR) with 2 CCP domains in *A*. *aegypti*, designated as AaSR-C, serves as a pattern recognition receptor to efficiently recognize DENV, and consequently recruits mosquito complement components to limit dengue replication [11].

*Aedes aegypti*, a member of the *Culicinae* subfamily, is a natural vector for Dengue and Yellow Fever viruses [1]. Several neurotropic flaviviruses, including WNV and JEV, have also been isolated in native *A*. *aegypti* or other *Aedes* species (http://www.cdc.gov/westnile/transmission/) [16]. Because these mosquitoes are easy to cultivate and the genome has been characterized, *A*. *aegypti* is an ideal insect model for viral pathogenesis and immune studies [17]. In this study, we have identified a *hig* homolog gene in *A*. *aegypti*, designated as *AaHig*. *AaHig* is highly expressed in the mosquito nervous system and enriched on the plasma membrane of neural cells. AaHig recognized DENV and JEV to directly interrupt flavivirus internalization into mosquito cells, therefore limiting flaviviral amplification in the mosquito brain. Immuno-blockade of AaHig resulted in a robust viral replication in mosquito brains, increased apoptosis of neural cells, and a dramatic reduction of the mosquito lifespan after flaviviral infection, suggesting that AaHig resists flavivirus spreading in the mosquito nervous system and therefore facilitates mosquito survival in the infection. Moreover, genetic or immune depletion of Hig homologue in *Culex pipien pallens* also significantly increased JEV infection, indicating Hig protein is functionally conserved in mosquitoes. Our study uncovered a previously unappreciated antiviral mechanism for Hig in the mosquito nervous system, which may provide insight into the sophisticated interactions between mosquito-borne viruses and the vector's antiviral immunity.

## Results

### Identification of a homolog of *Hig* in *A*. *aegypti*

**Fig 1:**
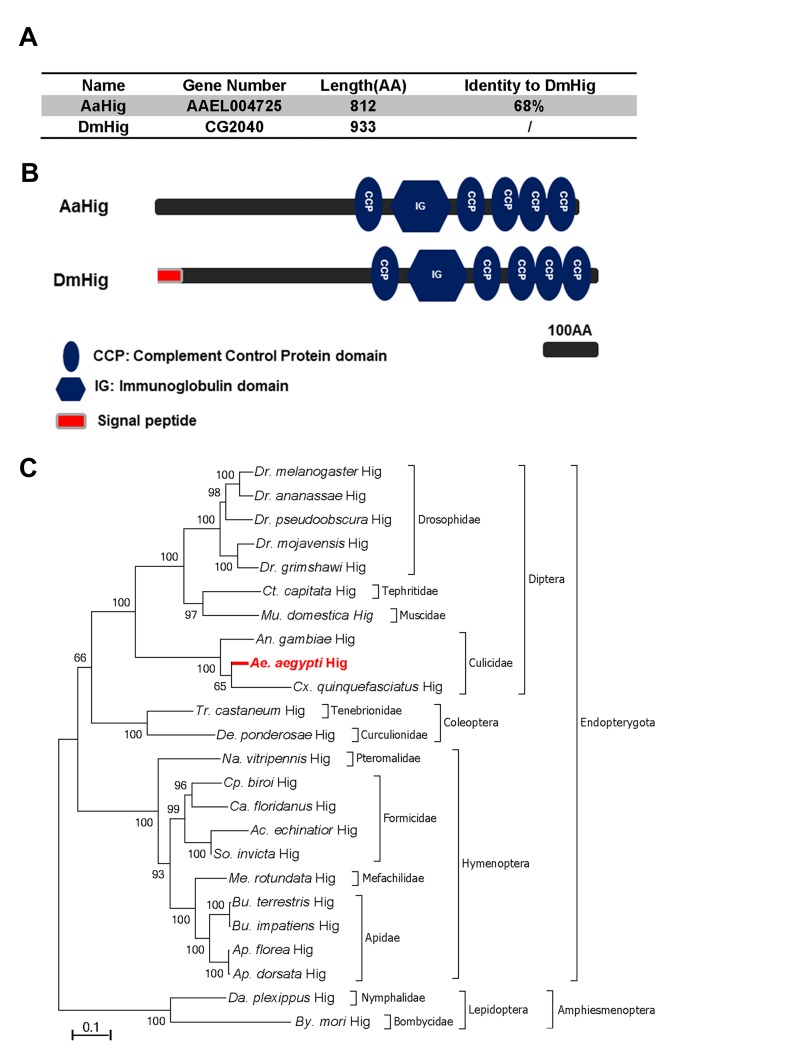
Bioinformatic comparison and phylogenetic analysis of *Hikaru genki* (*Hig*) in insects. (A) Percentage of amino acid identity between AaHig and *Drosophila melanogaster* Hig (DmHig). (B) Schematic representation of AaHig and DmHig. The functional modules were predicted using the SMART (http://smart.embl-heidelberg.de/smart/set_mode.cgi?GENOMIC=1) and Pfam (http://pfam.sanger.ac.uk/) websites. (C) Unrooted phylogenetic tree of insect Higs. The tree was constructed using the neighbour-joining (NJ) method based on the alignment of insect Hig protein sequences. The bootstrap values of 5000 replicates are indicated on the branch nodes. *Drosophila* spp. (*Dr*.); *Ceratitis* spp. (*Ct*.); *Musca* spp. (*Mu*); *Anopheles* spp. (*An*.); *Aedes* spp. (*Ae*.); *Culex* spp. (*Cx*); *Tribolium* spp. (*Tr*.); *Dendroctonus* spp. (*De*.); *Nasonia* spp. (*Na*.); *Cerapachys* spp. (*Cp*.); *Camponotus* spp. (*Ca*.); *Acromyrmex* spp. (*Ac*.); *Solenopsis* spp. (*So*.); *Megachile* spp. (*Me*.); *Bombus spp*. (*Bu*.); *Apis spp*. (*Ap*.); *Danaus spp*. (*Da*.); *Bombyx spp*. (*By*.).

The complement control protein (CCP) domain is an evolutionarily conserved module that is essential for complement function [18] by mediating the protein-protein interactions of complement components and recognition of pathogenic microorganisms [11,12,13,19]. Our previous work has identified a group of 10 proteins with CCP domains in *A*. *aegypti*, 6 of which play a role in the control of Dengue and Yellow Fever viruses infection of mosquitoes [11], indicating a general antiviral role for the *CCP* genes of *A*. *aegypti*. We next characterized these six genes by sequence comparison. Herein, a *CCP* gene, *AAEL004725*, was identified as a homologue of the *Drosophila* neural factor *Hikaru genki* (*DmHig*) (Fig 1A). The *AAEL004725*-encoded protein is predicted to contain 5 CCP domains and 1 immunoglobulin (Ig) domain, which is identical to those of *Drosophila Hig* (Fig 1B). We therefore designated *AAEL004725* as *A*. *aegypti Hig* (*AaHig*) throughout this investigation. The genomic comparison showed that the *Hig* genes are comprehensively expressed in insects, which are widely distributed throughout the orders of *Diptera*, *Coleoptera*, *Hymenoptera* and *Lepidoptera* (Fig 1C). However, no homolog was identified in other arthropods and vertebrates with available genomic information. The amino acid sequences of Hig proteins are evolutionarily conserved among various insect species, suggesting possible similar functions of these proteins.

### The specific expression of *AaHig* in the *A*. *aegypti* nervous system

**Fig 2:**
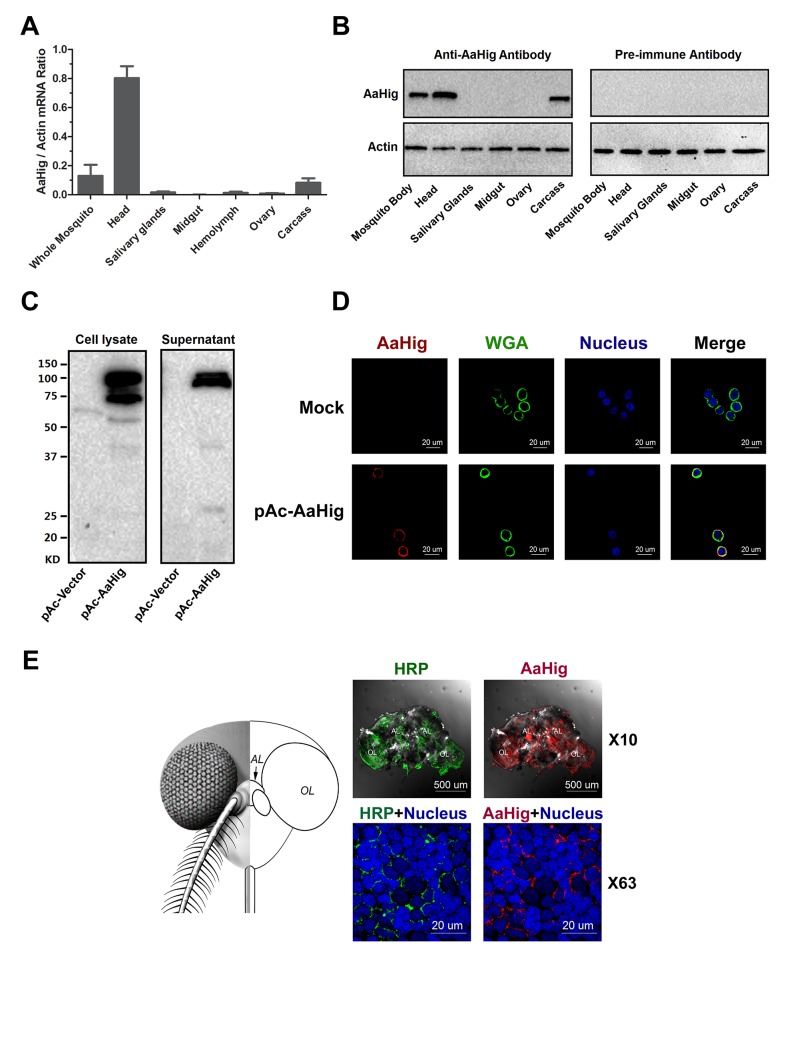
The high expression of AaHig in the brain of *A*. *aegypti*. (A-B) The expression of *AaHig* in various tissues of *A*. *aegypti*. The abundance of AaHig was assessed via SYBR Green qPCR (A) and immuno-blotting with an AaHig antibody (B). (A) The total RNA was isolated from mosquito tissues to determine *AaHig* expression by SYBR Green qPCR and normalized by *A*. *aegypti actin* (*AAEL011197*). The qPCR primers were described in the S1 Table. Data were represented as the mean ± standard error. (B) A variety of tissues were dissected from female *A*. *aegypti*. 50 μg of total protein from tissue lysates was loaded into each lane. The detection of *A*. *aegypti* actin was used as the internal control. (C) Secretory property of AaHig. The full length of AaHig gene (1bp-2436bp) was inserted into the expression vector pAc5.1/V5-His A with a V5 tag. The recombinant DNA plasmid (pAc-AaHig) was transfected into S2 cells. AaHig expression was detected with anti-V5 antibody in the cell lysate and supernatant. (D) AaHig staining in *A*. *aegypti* Aag2 cells. Aag2 is an *A*. *aegypti* cell lineage of embryonic origin. The pAc-AaHig recombinant plasmid was transfected into Aag2 cells. AaHig was stained by anti-V5 antibody and anti-mouse IgG Alexa-546 (Red). The plasma membrane was stained by the Wheat Germ Agglutinin (WGA) conjugated with Alexa-488 (Green). Nuclei were stained with To-Pro-3 iodide (Blue). Images were examined using the 63×objective lens of a Zeiss LSM 780 meta confocal. (E) AaHig is localized on the cell surface of neural cells of the *A*. *aegypti* brain. The brains were dissected from female *A*. *aegypti* for the *in situ* staining. An anti-HRP rabbit polyclonal antibody with anti-rabbit IgG Alexa-488 was used for surface staining of mosquito neural cells (Green). The AaHig protein was detected by an anti-AaHig mouse polyclonal antibody and anti-mouse IgG Alexa-546 (Red). Nuclei were stained blue with To-Pro-3 iodide. Images were examined using the 10× and 63×objective lens of a Zeiss LSM 780 meta confocal. AL, antennal lobes; OL, optic lobes.

*Drosophila* Hig is a secretory protein that is specifically expressed in the nervous system, which is an essential factor for the development of fly neural circuits [9,10]. We therefore assessed the abundance of *AaHig* in different tissues of *A*. *aegypti*. *AaHig* was highly expressed in the mosquito head, where the central nervous system is located. A mild level of *AaHig* abundance was also detected in the mosquito carcass (Fig 2A), which may come from the peripheral nervous system, e.g., the neural cells in the ventral nerve cords. To further understand the distribution of the AaHig protein, we expressed and purified a full-length peptide of AaHig in *Escherichia coli* (S1A Fig) and generated a polyclonal antibody in mice. The antibody was able to recognize S2-expressed recombinant AaHig protein (S1B Fig). Consistent with its mRNA expression pattern, the AaHig protein was detected specifically in mosquito heads and carcasses (Figs 2B and S2). We subsequently examined if *AaHig* expression is influenced by DENV infection. The infection did not change *AaHig* mRNA abundance in the investigated tissues at all evaluated time points (S3A–S3D Fig).

Unlike *Drosophila* Hig, that has a signal peptide [9], AaHig lacks a signal peptide at its N-terminus by sequence prediction (Fig 1B). To assess whether AaHig is secreted, we cloned the full-length AaHig (1-2436bp) into a pAc5.1/V5-His A expression vector, and the recombinant plasmid was subsequently transfected into *Drosophila* S2 cells. AaHig was strongly detected in the supernatant of transfected *Drosophila* S2 cells by Western-blotting (Fig 2C). Abundant AaHig was observed on the surface of transfected *A*. *aegypti* Aag2 cells (Fig 2D), indicating that AaHig is secreted and enriches on the plasma membrane (S4 Fig). To accurately measure the *in situ* localization of AaHig in the mosquito central nervous system, we stained the mosquito brains using an AaHig antibody. An anti-horseradish peroxidase (anti-HRP) antibody, used as a "pan-neuronal" label in insects to detect several epitopes on neuronal processes [20,21], was selected as a positive marker for the surface staining of mosquito neural cells. AaHig was detected comprehensively in both the antennal and optic lobes of mosquito brains and was enriched on the surface of neural cells (Fig 2E). To demonstrate the subcellular localization of AaHig, we collected brain tissues from female *A*. *aegypti* mosquitoes. The subcellular fractions, including the nucleus, mitochondria, cytoplasm and plasma membrane, were separated. Specific staining for AaHig was observed in the plasma membrane fraction (S5 Fig), further confirming the location of AaHig on the cell membrane of mosquito brain cells.

### The role of *Hig* in flavivirus infection of mosquitoes

**Fig 3:**
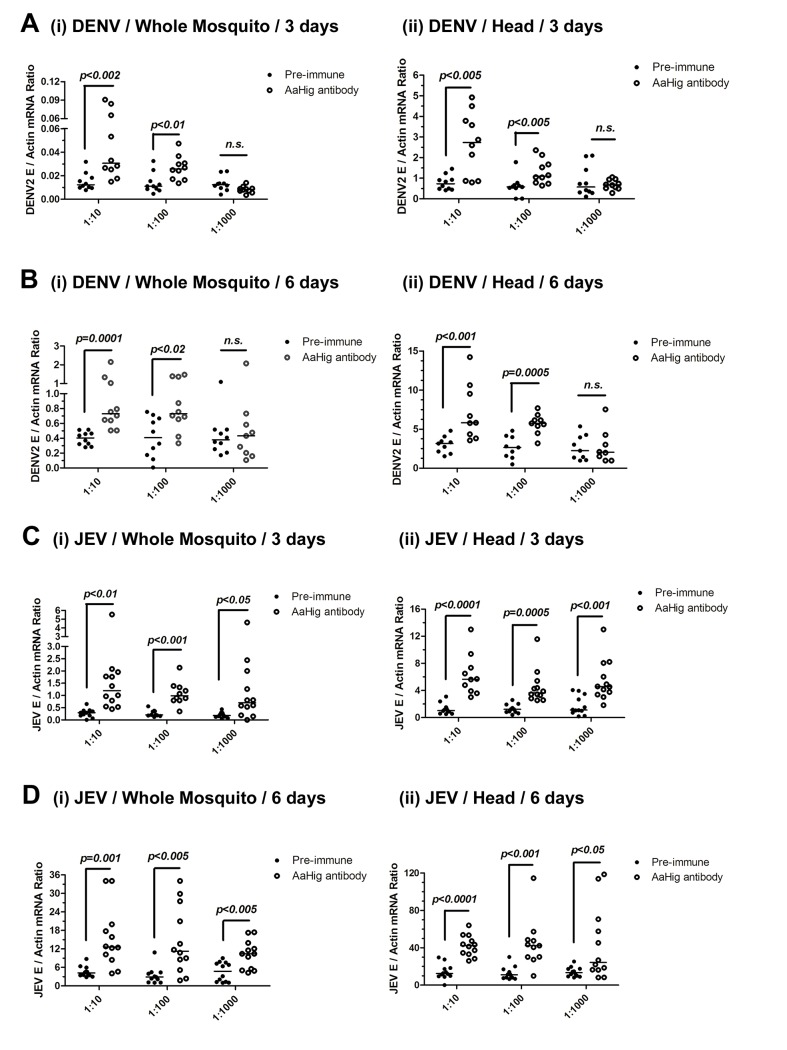
The role of *AaHig* in flaviviruses infection of *A*. *aegypti*. Immuno-blockade of AaHig enhanced the DENV-2 (A-B) and JEV (C-D) infections in the whole bodies (i) and heads (ii) of mosquitoes. The murine AaHig antibody, in the 10-fold serial dilutions, was premixed with 10 M.I.D._50_ viruses to co-microinject into the thorax of mosquitoes. The treated mosquitoes were sacrificed to examine the viral load in the whole mosquito bodies (i) and heads (ii) at 3 (A, C) and 6 (B, D) days post-infection by TaqMan qPCR and normalized against *A*. *aegypti actin*. The results were reproduced by 3 times. One dot represents 1 mosquito/head and the horizontal line represents the median of the results. The data were analyzed statistically using the non-parametric *Mann-Whitney* test.

dsRNA-mediated knockdown of *AaHig* significantly enhanced the DENV-2 and YFV burdens compared with that found in *green fluorescent protein* (*GFP*) mock dsRNA-treated mosquitoes, suggesting that *AaHig* is an antiviral factor in mosquitoes [11]. We reproduced the effect of dsRNA-mediated *AaHig* silencing on DENV-2 infection of *A*. *aegypti*. The intrathoracic inoculation of *AaHig* dsRNA significantly decreased *AaHig* expression in the whole mosquito bodies and heads at both the mRNA (S6A and S6B Fig) and protein (S6C Fig) levels. Three days after gene silencing, 10 M.I.D._50_ of DENV-2 were microinjected into the mosquitoes. The viral burden was assessed in whole bodies and heads via qPCR at 3 days and 6 days post-infection. In agreement with our previous observations [11], the knockdown of *AaHig* enhanced the DENV-2 burden in whole mosquitoes (S6D Fig) and heads (S6E Fig), confirming the important antiviral activity of *AaHig* in mosquitoes. To rule out any off-target effects of dsRNA-mediated RNA interference and further validate the role of *AaHig* in flaviviral infection, we immuno-blocked the native AaHig by thoracic inoculation of an AaHig antibody for functional investigation. To assess whether the antibody is delivered to the mosquito brain via its circulation system, we inoculated the diluted murine AaHig antibody into mosquito thoraxes, and subsequently tested its distribution in mosquito brains by *in situ* staining. The murine AaHig antibody was clearly detected on the surface of neural cells by fluorescence labeled anti-mouse IgG (S7 Fig), demonstrating the successful delivery of the AaHig antibody to the brain. Similar amount of the antibody was distributed in the brains of inoculated mosquitoes (S8 Fig). Therefore, serially diluted AaHig antibody was premixed with DENV-2 for mosquito microinjection. Neutralization of AaHig significantly enhanced DENV-2 infectivity of whole mosquitoes both 3 days (Fig 3A, i) and 6 days (Fig 3B, i) post-infection. Because AaHig is dominantly expressed in the central nervous system of mosquitoes (Fig 2E), we evaluated the viral burden in mosquito heads. Similarly, inoculation of AaHig antibody augmented viral replication in the mosquito heads on 3 and 6 days after infection (Fig 3A, ii and Fig 3B, ii). Through the approach of viral infection by oral feeding, immuno-blockade of AaHig also significantly enhanced the DENV burden in the whole bodies (S9A and S9B Fig) and heads (S9C and S9D Fig) of *A*. *aegypti*. AaHig antibody does not bind DENV-2 surface E protein or virions, indicating that increased viral load in mosquito heads was not a result of non-specific viral recognition by AaHig antibody. Furthermore, the viral burden in the salivary glands and the midgut was not influenced by the inoculation of AaHig antibody (S10 Fig), suggesting that AaHig is a neuron-specific factor that efficiently controls viral replication in the nervous system, regardless of the peripheral tissues.

**Fig 4:**
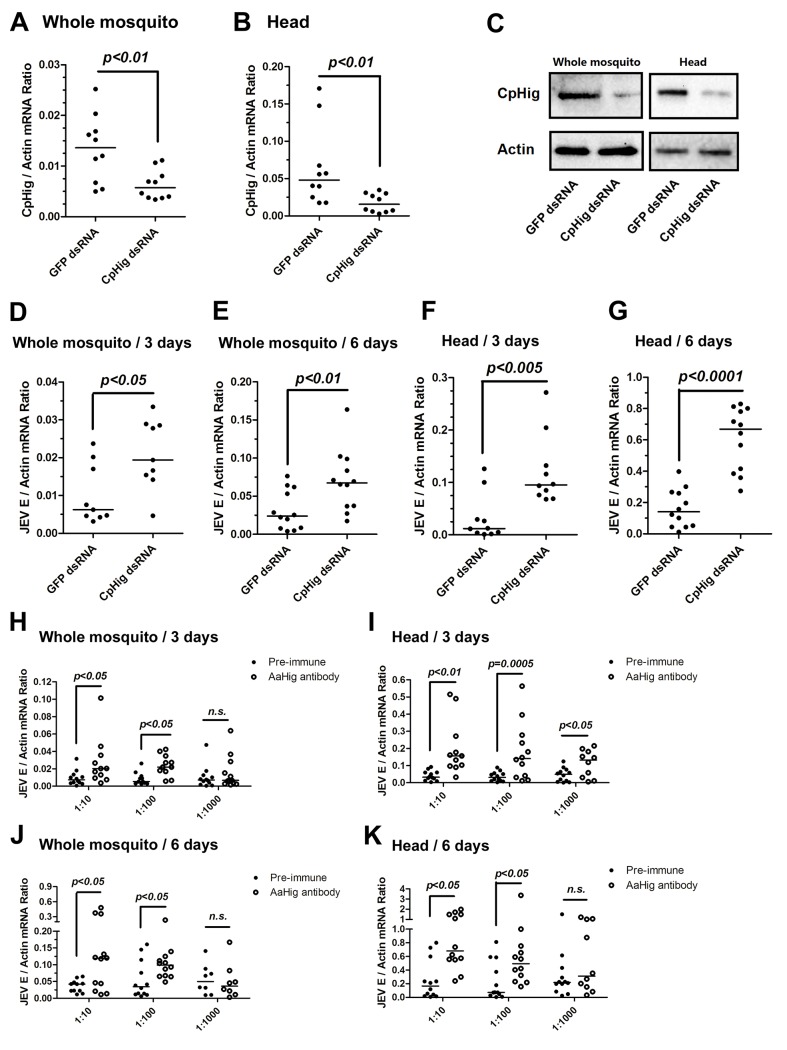
The antiviral effect of *Hig* in JEV infection of *C*. *pipiens pallens*. (A-C) Inoculation of *Culex pipiens pallens Hig* (*CpHig*) dsRNA significantly decreased the *CpHig* expression in the whole mosquitoes and heads of *C*. *pipiens pallens* at both the mRNA (A and B) and protein (C) levels. The *CpHig* abundance was assessed by SYBR Green qPCR (A and B) and immuno-blotting with an AaHig antibody (C) at 6 days post dsRNA microinjection. (D-G) Silencing *CpHig* enhanced JEV infection in *C*. *pipiens pallens*. 10 M.I.D._50_ JEV were inoculated at 3 days post *CpHig* dsRNA inoculation. The viral load of whole bodies (D and E) and heads (F and G) was assessed at 3 days and 6 days post-infection via Taqman qPCR and normalized with *Culex actin*. (H-K) Immuno-blockade of CpHig enhanced the JEV replication in *C*. *pipiens pallens*. The murine AaHig antibody, crossreacting with CpHig, was premixed with 10 M.I.D._50_ JEV to co-inoculate into the *Culex* mosquitoes thorax. The treated mosquitoes were sacrificed to examine the viral load in the mosquito bodies (H and J) and heads (I and K) at 3 and 6 days post-infection by TaqMan qPCR and normalized against *Culex actin*. (D-K) The primers and probes of Taqman qPCR were described in the S1 Table. The experiments were repeated 3 times with similar results. One dot represents 1 mosquito and the horizontal line represents the median of the results. The data were statistically analyzed by the non-parametric *Mann-Whitney* test.

Japanese encephalitis virus (JEV) is a mosquito-borne, neurotropic flavivirus that invades the central nervous system of vertebrates. JEV infection results in severe pathological and physiological damage to the human brain, high mortality and morbidity [1]. We therefore investigated the role of *AaHig* in JEV infection, using *A*. *aegypti* as a model. Compared to the pre-immune IgG, AaHig antibody treatment significantly enhanced the viral burden in both the whole bodies and heads on 3 (Fig 3C) and 6 (Fig 3D) days post-infection in *A*. *aegypti*. We next determined the role of *Hig* homologue in JEV infection of its natural vector, *Culex* mosquito. The *Hig* homologue (*CpHig*, *KP780883*) from the *Culex pipiens pallens* cDNA library was isolated and silenced via dsRNA thoracic inoculation in *C*. *pipiens pallens*. The expression of *CpHig* was significantly reduced at both the mRNA (Fig 4A and 4B) and protein (Fig 4C) levels. Three days post-dsRNA treatment, JEV was microinjected into the mosquitoes and the viral load was assessed via Taqman qPCR 3 and 6 days post-infection. Consistent with the results of JEV infection of *A*. *aegypti*, the knockdown of *CpHig* significantly increased the JEV load in the whole bodies (Fig 4D and 4E) and heads (Fig 4F and 4G) of *C*. *pipiens pallens* mosquitoes. Moreover, the inoculation of the AaHig antibody, which can also react with CpHig (Fig 4C), significantly increased the JEV burden in the *C*. *pipiens pallens* bodies (Fig 4H and 4J) and heads (Fig 4I and 4K), suggesting that the Hig protein is functionally conserved in mosquitoes.

To test if the antiviral activity of AaHig can be extended to other mosquito-borne viruses, we chose Sindbis virus (SINV), a member of the alphavirus genus. SINV was originally isolated from *Culex* mosquitoes, but a large number of mosquito species, including *Aedes*, are also able to act as vectors for SINV transmission in nature [22]. The *A*. *aegypti*-SINV system has been used extensively as a model for understanding arbovirus-mosquito interactions [23,24,25]. We therefore intrathoracically inoculated *AaHig* dsRNA in *A*. *aegypti*. Three days post-gene silencing, 10 M.I.D._50_ of SINV were microinjected into the mosquitoes and the viral burden was assessed in whole bodies and heads via qPCR at 3 days and 6 days post-infection. However, the silencing of *AaHig* did not influence the SINV burden in whole bodies (S11A and S11B Fig) or mosquito heads (S11C and S11D Fig). Subsequent investigation demonstrated that AaHig did not interact with the SINV envelope proteins E1, E2 or E3 (S11E and S11F Fig), suggesting a flavivirus-specific antiviral role for AaHig.

### AaHig prevents flaviviral infection-induced neural apoptosis and lethality

**Fig 5:**
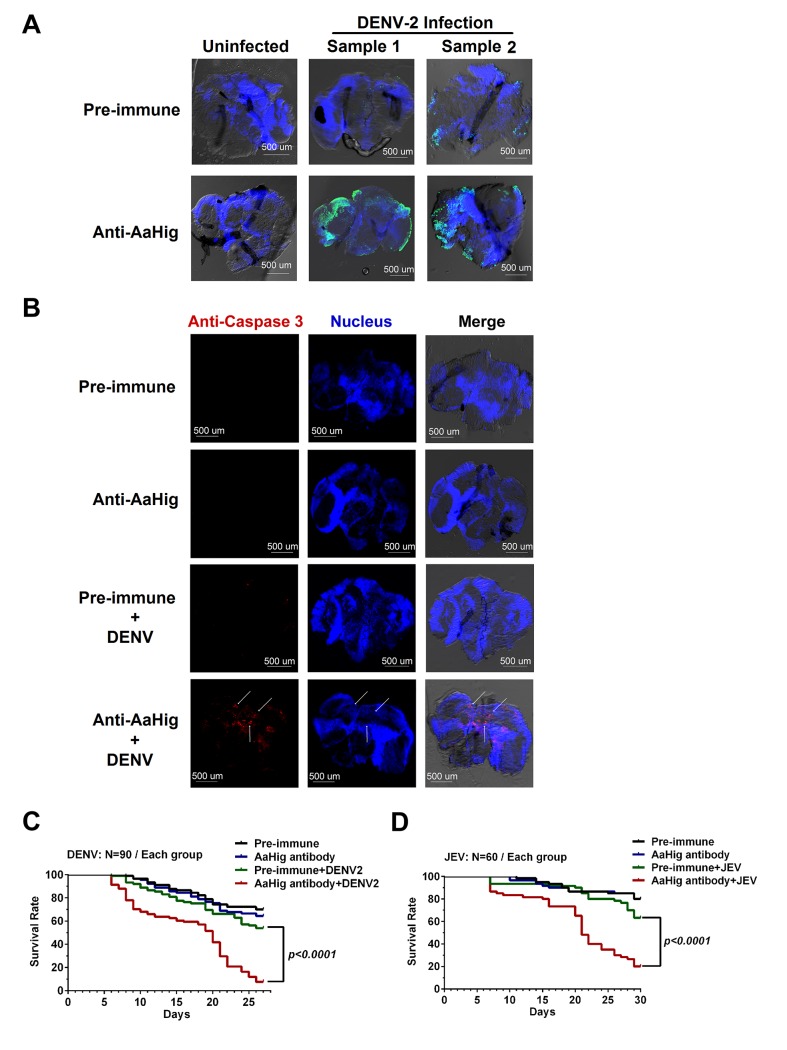
Blockade of AaHig resulted in apoptosis of neural cells and reduction of mosquito lifespan in DENV and JEV infections. (A) Detection of *in situ* cell apoptosis in mosquito brains. The apoptosis of mosquito tissues was determined by the terminal deoxynucleotidyl transferase (TdT)-mediated dUTP nick end-labeling (TUNEL) assay. Six days post DENV-2 infection with or without AaHig antibody treatment, the mosquito heads were cut off and fixed in 4% paraformaldehyde for the brains isolation. FITC filter was used to detect cell apoptosis (Green). The nuclei were stained with To-Pro-3 iodide (Blue). TUNEL-positive staining patterns were acquired by a Zeiss LSM 780 meta confocal microscope with a Multi-Track mode. (B) Measurement of apoptotic damage by a caspase-3 antibody staining. The preparation of mosquito brains has been described in (A). The apoptotic damage was stained by a cleaved caspase-3 antibody (Red), which was used as a marker for apoptosis in *Drosophila*. The nuclei were stained with To-Pro-3 iodide (Blue). The tissues were imaged using the 10×objective lens of a Zeiss LSM 780 meta confocal. (C-D) Blockade of AaHig reduced the mosquito survival in DENV-2 (C) and JEV (D) infections. 10 M.I.D._50_ DENV-2 or JEV were premixed with the 10-fold diluted AaHig antibody for mosquitoes microinjection respectively. Pre-immune antibody with the viruses and the antibodies without viruses served as the mock controls. The infected mosquitoes were maintained with standard rearing and daily observed for calculation of survival rate. The data were statistically analyzed by the *Log-rank* (*Mantel-Cox*) test.

The mosquito lifespan is rarely decreased by persistent flavivirus infection [5,6,7,8]. The antiviral factors in mosquitoes are unable to eradicate viruses from mosquitoes, but can limit the viral burden to a tolerable level that does not elicit significant tissue damage. Without these antiviral mechanisms, viral replication could cause damage to the mosquito physiology and therefore decrease the mosquito lifespan [26]. Previous studies have suggested that arboviral infection leads to apoptosis in mosquito tissues. The midgut epithelial cells of *Culex pipiens* showed apoptosis following infection by WNV [27]. Apoptosis of the tissues of *Culex quinquefasciatus* was observed after WNV infection [28]. Moreover, the level of apoptosis is correlated with viral persistent infection in mosquitoes [29,30]. We therefore assessed apoptosis via TUNEL staining in DENV-2 infected mosquito brains with or without AaHig antibody treatment. The number of apoptotic cells in the brains of AaHig antibody/DENV-2-treated mosquitoes was greater than that in the mock controls (Fig 5A). Additionally, the more severe apoptotic damage in the AaHig-depleted mosquito brains was determined by a cleaved caspase-3 antibody for detection of apoptosis in *Drosophila* [31] (Fig 5B). We consequently assessed whether immuno-blockade of AaHig influences mosquito survival upon flavivirus infection. 10 M.I.D._50_ DENV-2 or JEV were premixed with the diluted AaHig antibody for mosquito microinjection, respectively. Pre-immune antibody with the viruses and the antibodies without viruses served as mock controls. Consistent with previous studies, DENV infection was not lethal to mosquitoes when compared to mock infection. However, the infection became lethal when AaHig function was blocked with an antibody (Fig 5C). Similar results were observed with JEV infection (Fig 5D). These data clearly demonstrate that AaHig is a key factor for mosquito survival during persistent flavivirus infection.

### The antiviral activity of AaHig depends on its direct interaction with flaviviral virions

**Fig 6:**
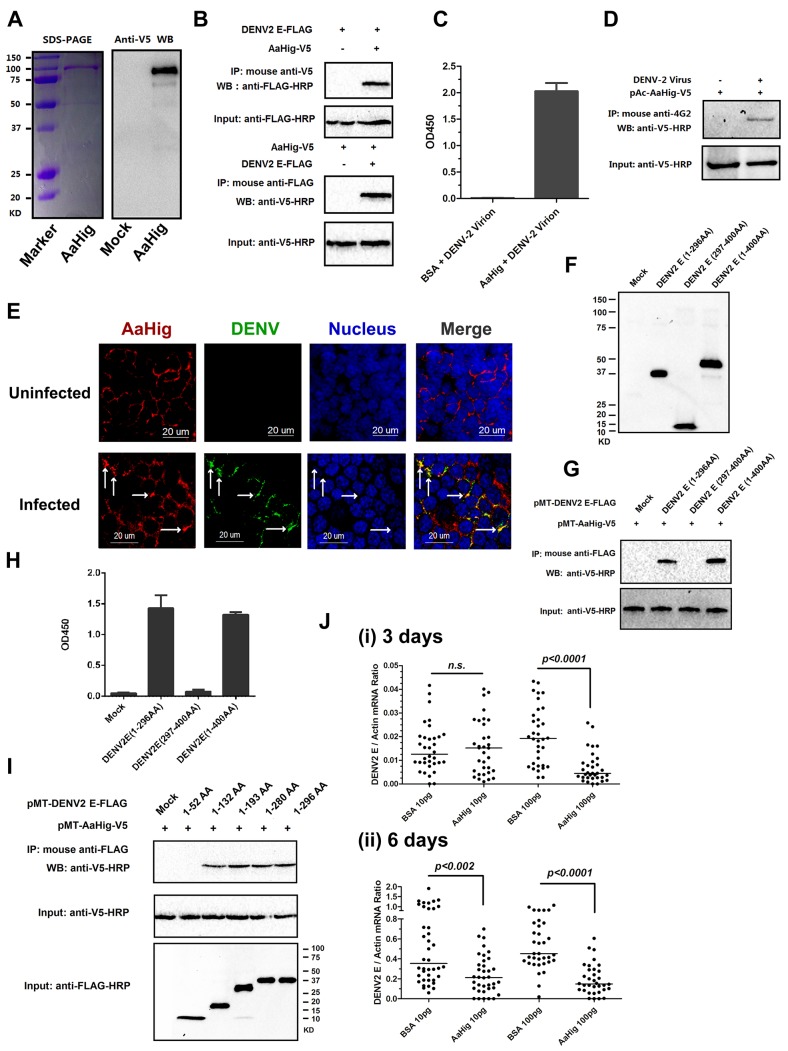
AaHig directly recognizes dengue virus. (A) Expression and purification of AaHig from *Drosophila* S2 cells. The full length *AaHig* was cloned into the pMT/BiP/V5-His A expression vector. The recombinant plasmid was transfected into *Drosophila* S2 cells, and the expression was probed using an anti-V5 mAb. The supernatant from mock-transfected S2 cells was used as the mock control (Right Panel). Recombinant AaHig protein, produced in *Drosophila* cells, was purified using a Ni-His column (Left Panel). (B) AaHig interacted with DENV-2 E protein in a co-immunoprecipitation (co-IP) assay. The purified AaHig (V5) and DENV-2 E (FLAG) proteins were used to investigate the interaction of the proteins. We reproduced the experiment 3 times. (C) AaHig captured DENV-2 virions in an ELISA assay. The binding was probed using the flavivirus E mAb 4G2. The data were presented as the mean ± standard error. The experiment was reproduced 3 times. (D) AaHig interfaced with DENV virions in the infected mosquito cells. pAc-AaHig was transfected in Aag2 cells, and subsequently the cells were infected by 5 M.O.I. DENV-2 at 12 hrs post transfection. The uninfected cells transfected by pAc-AaHig were used as a mock control. After 48 hrs infection, the cells were lysated and an anti-flaviviral E 4G2 mAb was added into the lysate for the pull-down assay. We reproduced the experiment 3 times. (E) The co-staining between AaHig and DENV-2 in the *A*. *aegypti* brain. The mosquito brains were dissected from uninfected mocks and infected mosquitoes at 6 days post infection to undergo immunofluorescence staining. AaHig was stained with anti-mouse IgG Alexa-546 (Red), and the DENV-2 E protein was stained using anti-human IgG Alexa-488 (Green). Nuclei were stained with To-Pro-3 iodide (Blue). Images were examined using the 63×objective lens of a Zeiss LSM 780 meta confocal. (F) The expression of ectodomains in the DENV-2 E protein. The genes of ED1+ED2 (1-296AA) and ED3 (297-400AA) of DENV-2 E protein were cloned into pMT/BiP/V5-His A vector and the encoded peptides were expressed in *Drosophila* S2 cells. The supernatant from empty vector-transfected S2 cells was used as a mock. The recombinant peptides were detected with an anti-FLAG antibody via western blotting. (G) The ectodomains of DENV-2 E protein interacted with AaHig in a co-IP assay. The protein complex was pulled down with an anti-FLAG antibody and detected using an anti-V5-HRP antibody. We reproduced the experiment 3 times. (H) The ectodomains of DENV-2 E protein interfaced with AaHig by an ELISA assay. The binding was probed using an anti-V5-HRP antibody. The data were presented as the mean ± standard error. The experiment was reproduced 3 times. (I) The interaction between AaHig and the linear motifs of ED1/ED2. The linear motifs of ED1 and ED2 were sequentially deleted from the ectodomains. The five truncations were cloned into the pMT/BiP/V5-His A vector, and subsequently expressed in the S2 cell supernatant. The protein complex was pulled down with an anti-FLAG antibody and detected using an anti-V5-HRP antibody. We reproduced the experiment 3 times. (J) Inoculation of AaHig impaired DENV-2 infectivity in *A*. *aegypti*. Two laddered concentrations of purified AaHig protein were premixed with 10 M.I.D._50_ of DENV-2 for mosquito thoracic microinjection. The infected mosquitoes were sacrificed at 3 (i) and 6 (ii) days post infection. The viral load was determined by Taqman qPCR and normalized by *A*. *aegypti actin*. The results were combined from 3 independent experiments. One dot represents 1 mosquito and the horizontal line represents the median value in the figures. The statistical analysis was done with the *Mann-Whitney* test.

The CCP domain functions as a viral recognition module [11,12,13,15,19]. We next tested if AaHig employs a similar strategy to control viral infections. The full-length AaHig protein was generated and purified in a *Drosophila* S2 cell expression system (Fig 6A). The purified AaHig protein strongly interacted with the DENV-2 E protein in a co-IP assay (Fig 6B) and captured DENV-2 virions efficiently by ELISA (Fig 6C). Moreover, DENV-2 virions were pulled down by ectopically expressed AaHig protein in the infected mosquito Aag2 cells (Fig 6D). We next performed immunofluorescence staining of the mosquito brains to explore whether native AaHig interacts with DENV-2 particles. Co-staining of AaHig and DENV was clearly observed on the surface of mosquito neural cells (Fig 6E). The interaction between AaHig and JEV was also measured by ELISA (S12A Fig) and co-IP (S12B Fig) assays. We next investigated the details of flavivirus-AaHig interaction. The length of the Dengue E protein is approximately 500 amino acids, of which the N-terminal 400 amino acids form an ectodomain. The ectodomain consists of three domains that are referred to as envelope protein domain I (ED1), ED2, and ED3 [32]. Both ED1 (1-52AA; 133AA-193AA; 281-296AA) and ED2 (53-132AA; 194-280AA) are structural domains, and the linear motifs of ED1 and ED2 are interlaced. However, the ED3 domain consists of an entirely linear sequence (297AA-400AA) [32]. Based on these defined domains, we first cloned and expressed two truncated peptides in *Drosophila* S2 cells: the ED1+ED2 (1-296AA) and ED3 (297-400AA) peptides (Fig 6F). AaHig strongly interacted with the ED1+ED2 peptide, but no interaction was detected between AaHig and the ED3 (Fig 6G and 6H) peptide. To further determine the binding motifs in ED1 and ED2, we next constructed five truncations in which the linear motifs were sequentially deleted from the ED1 and ED2 domains (Fig 6I). The results indicated that the 53 AA-132 AA motif in ED2 is indispensable for the interaction with AaHig (Fig 6I). We then assessed whether the 53 AA-132 AA motif is highly conserved among flaviviruses. The motif sequence shares 46%-75% identity among the E proteins of DENV, JEV and YFV. Computational structure modeling also demonstrated that the 53 AA-132 AA motif is conserved among the three flaviviruses, suggesting that a consistent binding mechanism exists between AaHig and different flaviviruses. To examine the physiological relevance of AaHig and DENV binding, the purified AaHig protein together with DENV-2 virions was microinjected into *A*. *aegypti* and the DENV loads were assessed. Inoculation of AaHig significantly impaired DENV-2 infectivity on 3 (Fig 6J, i) and 6 days (Fig 6J, ii) post-infection, validating an important antiviral role for AaHig in mosquitoes.

**Fig 7:**
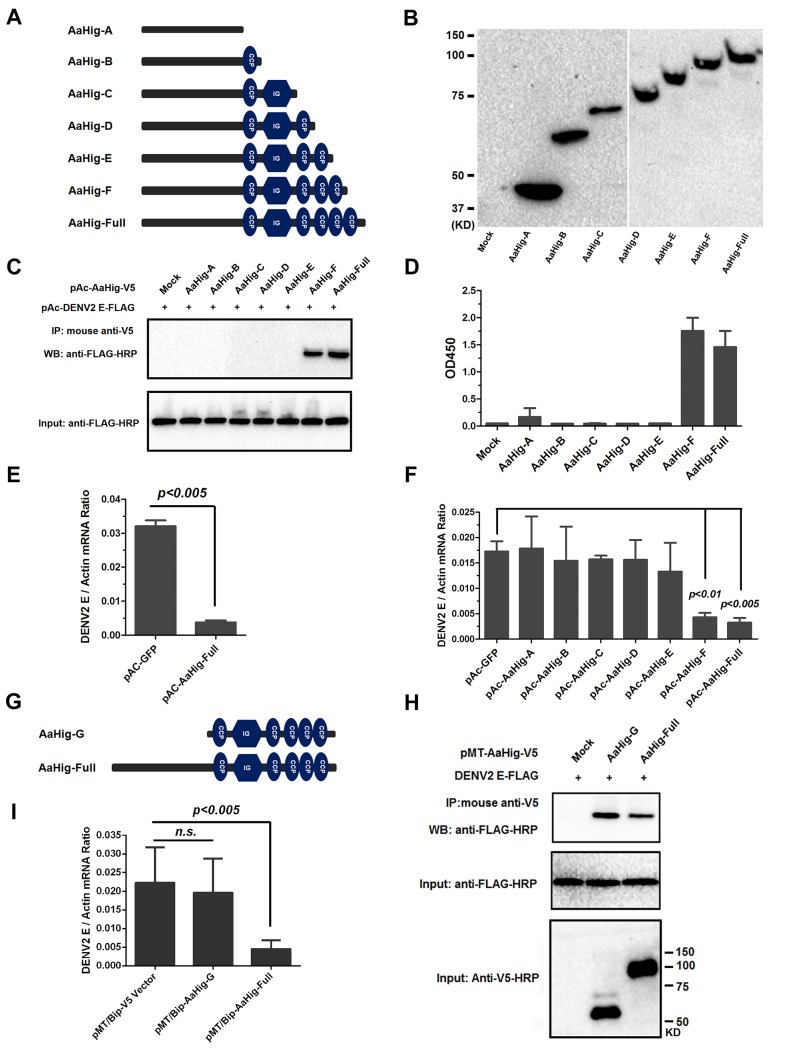
Both the viral interaction and membrane bound of AaHig are essential for the AaHig antiviral activity. (A-B) Construction and expression of recombinant AaHig truncations. The schematic representation of AaHig truncations with sequential depletion of modules is shown in the panel A. The fragments of *AaHig* truncated genes were constructed into pAc5.1/V5-His A vector to express in *Drosophila* S2 cells (pAc-AaHig-A ~ pAc-AaHig-F). The S2 cells transfected by the empty vector were used as a mock control. The expression in the supernatant was detected by western blotting with anti-V5 mAb (B). (C) The interaction between AaHig truncated peptides and the DENV-2 E protein in a co-IP assay. The cell supernatant was individually incubated with purified DENV-2 E protein. The supernatant from the pAc-GFP transfected cells was used as a mock control. The protein complex was pulled down with an anti-V5 antibody and probed using an anti-FLAG-HRP antibody. The experiment was reproduced 3 times. (D) The detection of binding capacity between AaHig fragments and DENV-2 E protein by ELISA. The supernatant of pAc-AaHigs and pAc-GFP transfected cells was used to assess the binding activity with DENV-2 E proteins. The binding was probed using the anti-V5 mAb. The data were presented as the mean ± standard error. The experiment was repeated 3 times with the similar result. (E) AaHig resists DENV-2 infection in *A*. *aegypti* Aag2 cells. The pAc-AaHig-Full recombinant plasmid was transfected into Aag2 cells. The pAc-GFP was used as negative control. After 48 hrs, the 0.01 M.O.I. DENV-2 was added into the cells and the viral load was determined by Taqman qPCR and normalized by *A*. *aegypti actin*. The data were presented as the mean ± standard error. The result was combined from 3 independent experiments. (F) The role of AaHig truncated peptides in DENV-2 infection of Aag2 cells. A variety of AaHig truncations were expressed in Aag2 cells. After 0.01 M.O.I. DENV-2 infection, the viruses were determined by Taqman qPCR and normalized against *A*. *aegypti actin*. The data were presented as the mean ± standard error. The result was combined from 2 independent experiments. (G) The schematic representation of AaHig-G truncation. (H) The interaction between AaHig-G peptide and the DENV-2 E protein in a co-IP assay. The gene of *AaHig-G* was cloned into pMT/BiP/V5-His A and expressed in S2 cells. The cell supernatant with AaHig-G was individually incubated with purified DENV-2 E protein. The supernatant from the pMT/BiP/V5-His A vector transfected cells was used as a mock control. The transfection of pMT-AaHig-Full served as a positive control. The protein complex was pulled down with an anti-V5 antibody and probed using an anti-FLAG-HRP antibody. The experiment was reproduced 3 times. (I) The role of AaHig-G in DENV-2 infection of Aag2 cells. Both *AaHig-G* and *AaHig-Full* cloned in pMT/BiP/V5-His A vector were ectopically expressed in S2 cells. The conditional medium premixed with 0.01 M.O.I. DENV-2 was added into mosquito Aag2 cells for infection. The infectivity were determined by Taqman qPCR and normalized against *A*. *aegypti actin*. The data were presented as the mean ± standard error. The result was combined from 3 independent experiments.

We next evaluated the importance of the conserved functional domains of AaHig for its antiviral activity. Six deletion mutants were constructed into the pAc5.1/V5-His A vector and expressed in *Drosophila* S2 cells (Fig 7A and 7B). Both co-IP and ELISA assays showed that deletion of the second C-terminal CCP module (680 AA-742 AA) abrogated the interaction between the AaHig and DENV-2 E proteins, suggesting that this module is necessary for the protein-virus recognition (Fig 7C and 7D). Aag2 is an *A*. *aegypti* cell lineage of embryonic origin [33] that is permissive to many mosquito-borne flaviviruses [34,35], and is thus used as a model for the study of mosquito immunity and viral pathogenesis. However, Aag2 cells did not express AaHig. We then determined the impact of transient expression of AaHig on viral infectivity. The DENV burden was significantly decreased in *AaHig*-transfected Aag2 cells in comparison with that in pAc-GFP transfected cells (Fig 7E). Notably, the truncations that retained the ability to interact with the DENV2 E protein (AaHig-F and AaHig-Full) all showed a significant antiviral activity, while the truncations (AaHig-A ~ AaHig-E) that failed to bind DENV-E lost antiviral activity (Fig 7F). These results indicate that the binding capacity between AaHig and viruses is essential for the antiviral activity of AaHig in mosquito cells.

### The antiviral activity of AaHig requires its plasma membrane localization

Since AaHig is secreted and enriched on the plasma membrane of neural cells, we next investigate the role of membrane localization in the antiviral activity of AaHig. Six truncations (Fig 7A) in which the functional domains of AaHig were sequentially deleted were constructed in the pAc5.1/V5-His A vector and expressed in mosquito Aag2 cells. Immunofluorescence staining indicated that these AaHig truncations were localized to the surface of mosquito cells (S13 Fig), suggesting that the N-terminal domain (located in AaHig-A) may be responsible for anchoring AaHig in the membrane. We therefore constructed a deletion mutant that lacked the N-terminal sequence (AaHig-G, 323 AA-812 AA) (Fig 7G). AaHig-G retained the ability to bind to the DENV E protein (Fig 7H); however, AaHig-G failed to localize to the plasma membrane of mosquito cells (S13 Fig). As a result, AaHig-G exhibited no antiviral activity (Fig 7I), indicating that the antiviral activity of AaHig also relies on its proper association with the plasma membrane.

AaHig is a resistant factor against Dengue infection in *A*. *aegypti* cells (Fig 7E). To investigate whether the antiviral mechanism of AaHig is specific to mosquito cells, we assessed the role of AaHig in DENV-2 infection of A549 (a human alveolar basal epithelial cell line) and Vero (an African green monkey kidney epithelial cell line) cells. The presence of AaHig failed to reduce the DENV-2 burden in the mammalian cells (S14A and S14B Fig). Because *Drosophila* S2 cells are susceptible to DENV infection [36], we determined the effect of *Drosophila melanogaster Hig* (*DmHig*) on DEVN-2 infection of *Drosophila* cells. *DmHig* was cloned and expressed in S2 cells (S14C Fig). The overexpression of *DmHig* significantly impaired DENV-2 infectivity in both *A*. *aegypti* Aag2 (S14D Fig) and *Drosophila* S2 (S14E Fig) cells, indicating that the antiviral mechanism of Hig proteins is specific to and conserved in insects.

The antiviral activity of AaHig relies on proper localization to the cellular membrane (Figs 7I and S13). To determine the reason for the lack of AaHig antiviral activity in mammalian cells (S14A and S14B Fig), we determined the ability of AaHig to bind to the surface of Vero cells. AaHig cannot coat on Vero cells, as indicated by an immuno-staining assay (S15 Fig), suggesting that mammalian cells may not express the membrane receptor(s) for AaHig anchoring. These results explain why AaHig exerts no antiviral activity in mammalian cells.

### AaHig limits flavivirus infection by interrupting endocytic viral entry

In the absence of adaptive immunity, mosquitoes employ sophisticated innate immune machineries to detect and limit invading viruses, including RNA interference (RNAi) [37,38], antimicrobial peptides (AMPs) [11,39], reactive oxygen species (ROS) [40] and melanization [41,42]. To uncover the antiviral mechanism of AaHig, we investigated the role of AaHig in the above-mentioned mechanisms. Immuno-blockade of AaHig by antibody inoculation did not alter the expression of the *AMP* genes, RNAi-related genes (*Ago2* and *Dicer2*), or ROS-related genes (*Duox1*, *Duox2*) in mosquitoes (S16 Fig). Overexpression of *AaHig* in Aag2 cells did not influence the melanization activity (S17A and S17B Fig), and knockdown of *AaHig* was also ineffective for H_2_O_2_ release in various mosquito parts (S17C Fig). These results indicate that the antiviral activity of AaHig is independent of the known innate immune pathways.

**Fig 8:**
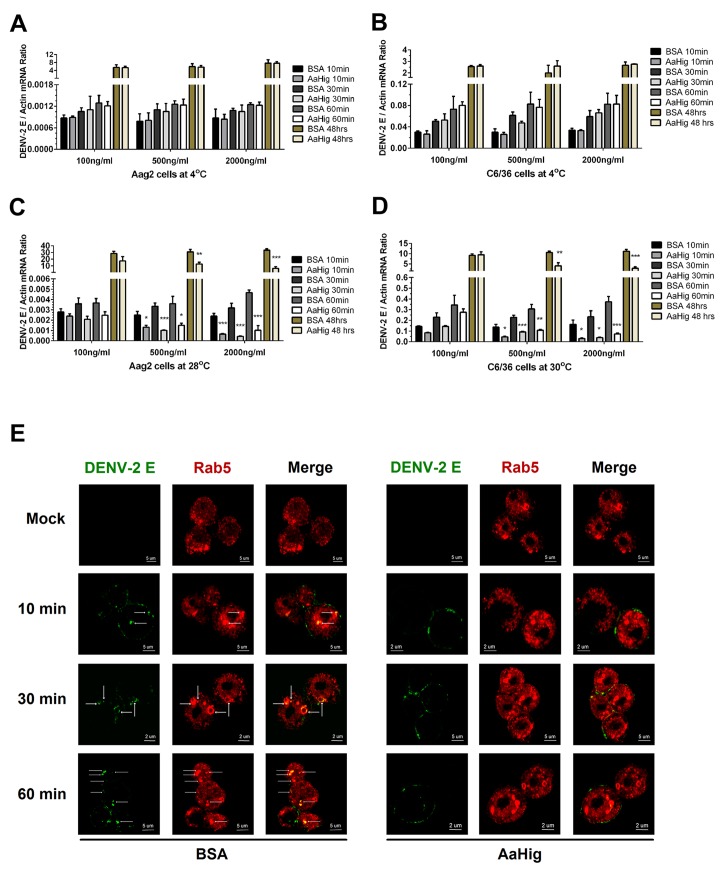
AaHig interrupts flaviviral endocytosis into mosquito cells. (A-B) Viral attachment assay. The serial concentration of purified AaHig protein was premixed with 5 M.O.I. DENV-2 on ice, and then the Aag2 (A) and C6/36 (B) cells were consequently preadsorbed with the mixture for a time course at 4oC. The cells were washed 5 times by cold PBS and collected at certain time points for total RNA isolation. (C-D) Viral entry assay. The serial concentration of purified AaHig protein was premixed with 5 M.O.I. DENV-2 on ice, and then the mixture was transferred into the cultured Aag2 cells (C) at 28°C and C6/36 (D) cells at 30°C respectively. The cells were stringently washed 5 times by PBS at room temperature, and then collected at serial time points for detection. (A-D) For the assay at 48 hours, the cells were washed 5 times after 1 hr incubation at 4°C (A and B) or 28°C /30°C (C and D), and consequently cultured at 28°C (Aag2) or 30°C (C6/36) for an additional 48 hrs. The viral genome was determined by Taqman qPCR and normalized by *A*. *aegypti actin*. The data were presented as the mean ± standard error. The result was combined from 3 independent experiments. *, *p<0*.*05*; **, *p<0*.*01*; ***, *p<0*.*001*. (E) Detection of AaHig-mediated entry interruption by structured illumination microscopy imaging. The Aag2 cells were seeded in a class bottle cell culture dish. The purified AaHig protein premixed with 10 M.O.I. DENV-2 was added into the cells. The equal amount of BSA was used as a negative control. The treated cells were collected and analyzed at a time course. DENV-2 was stained by the 4G2 mouse mAb and anti-mouse IgG Alexa Fluor-488 (Green). The early endosome marker, Rab5, was stained by a rabbit polyclonal antibody and anti-rabbit IgG Alexa Fluor-546 (Red). Images were examined using the 100×objective lens of a Zeiss ELYRA PS.1 structured illumination microscopy.

AaHig is enriched on the surface of neural cells (Fig 2E), and also directly interacted with the surface envelope proteins of flaviviruses. Both the viral binding and membrane anchoring were necessary for the antiviral activity of AaHig (Fig 7F and 7I). We therefore hypothesized that AaHig binding to flaviviruses may directly interrupt viral entry into mosquito cells. First, we assessed whether AaHig influenced flavivirus attachment on the surface of Aag2 and C6/36 cells. When virions are added to cells at 4°C, the virions are just tethered on the cell surface, but are not internalized [43,44,45]. After five washes with cold PBS buffer, the cells were collected to measure the viral burdens by qPCR. Compared to the mock control, incubation of AaHig did not block DENV-2 attachment onto the surface of Aag2 (Fig 8A) and C6/36 (Fig 8B) cells. Consistent results were also observed for JEV attachment to Aag2 (S18A Fig) and C6/36 (S18B Fig) cells. We next investigated the role of AaHig in viral internalization. Viruses and the proteins were incubated with the cells at 28°C (Aag2) or 30°C (C6/36) for various times, and viral loads were quantified after a stringent wash. AaHig blocked DENV-2 (Fig 8C and 8D) and JEV (S18C and S18D Fig) entry into the mosquito Aag2 and C6/36 cells at all time points post incubation, suggesting that AaHig reduces flaviviral replication by directly interrupting viral internalization into mosquito cells.

DENV enters mosquito cells through clathrin-dependent endocytosis. The viral particles are then transported to endosomes, where the viral genome is released into the cytoplasm, leading to successful infection [43]. The entry processes can be dissected into (i) viral recognition by cellular attachment factors/receptors, (ii) viral entry via endocytosis, and (iii) viral membrane fusion with endosomal membranes, leading to the release of the viral genome into the cytoplasm [46,47]. Our data demonstrated that AaHig does not interrupt DENV attachment to the mosquito cell membrane (Fig 8A and 8B); however, AaHig significantly blocks the internalization of DENV into mosquito cells (Fig 8C and 8D), suggesting the AaHig inhibits viral entry. We next determined whether AaHig interferes with the endocytic transport of DENV. The AaHig protein was premixed with DENV-2 and subsequently incubated with Aag2 cells at 28°C for a time course. The same amount of BSA mixed with DENV-2 was used as a mock control. The viral particles were stained and observed using high-resolution structured illumination microscopy (SIM). The GTPase Rab5 was used as a specific marker for early endosomes [43,48]. In the control cells, the viruses were rapidly internalized into early endosomes. In contrast, most of the viral particles in the AaHig-treated cells were retained on the cell surface (Fig 8E), suggesting that the AaHig protein interferes with early viral endocytosis. To determine whether AaHig is a general inhibitor of endocytic pathway or it specifically directly retains flaviviral particles on the cell surface to prevent viral entry, we used nano-beads with a 10–100 nm diameter, which mimic the size of the viruses, to perform a particle uptake assay in mosquito Aag2 cells. AaHig exhibited no nonspecific interactions with the beads. The number of internalized beads was measured using flow cytometry. Compared to the control groups, AaHig did not reduce particle uptake (S19 Fig), indicating that AaHig does not interfere with general endocytosis. AaHig doesn’t interface with the SINV envelope proteins (S11F Fig). We next explored the role of AaHig in Sindbis entry of human alveolar basal epithelial A549 and mosquito Aag2 cells. However, AaHig did not influence the Sindbis burden in either type of cells in the viral entry assay (S20 Fig). In summary, AaHig specifically binds to flaviviral particles, this physical interaction does not interrupt viral attachment or binding to cellular receptors but inhibits receptor-mediated endocytosis.

## Discussion

The central nervous system plays a predominant role in organisms associated with cognition and higher-order functions, which is key to their successful survival. Many mosquito-borne flaviviruses particularly invade the central nervous system in vertebrates, resulting in dramatic neural degeneration and damage. As natural vectors, mosquitoes are highly permissive to flaviviral infection, and yet they maintain a normal physiology and life span [5,6,7,8]. The mosquito’s brain, similarly to the mammalian brain, is sensitive to viral infection [4], and thus its antiviral machinery is supposedly very efficient in limiting the viral burden to a safe level [5,6]. However, little is known about the neuron-specific antiviral mechanism in mosquitoes. In this study, we report that AaHig controls viral replication specifically in the nervous system by interfering with viral entry, and its activity prevents lethal flaviviral infection of mosquitoes.

Both mammals and insects are equipped with sophisticated immune systems to detect and eliminate invading pathogens before they cause significant physiological damage. Viral recognition by host factors is an essential process for the antiviral response. The complement control protein (CCP) domain is evolutionarily conserved and interfaces with the surface of several pathogenic viruses [18]. We have identified 6 *CCP* genes against DENV and YFV infections of *A*. *aegypti*, in which a scavenge receptor (AaSR-C) with 2 CCP modules recognizes DENV and the complement component to exert potent anti-DENV activity [11]. In the current study, we found that a *CCP* factor named *AaHig* is specifically expressed in the nervous system of *A*. *aegypti*. AaHig is a secretory factor localized to the surface of neural cells. Immuno-blockade of AaHig significantly enhanced flaviviral replication in mosquito heads, and AaHig was capable of efficiently capturing flaviviral particles *in vivo* and *in vitro*, therefore directly interrupting viral entry into mosquito cells to limit viral replication. The antiviral activity of Hig homologue was also observed in JEV infection of *C*. *pipien pallens*. Both the virus-binding and membrane-targeting ability of AaHig were essential for its antiviral activity. Indeed, the direct blockade of viral entry is a common strategy against viral invasion of mammalian cells. A siRNA-mediated gene silencing screen has identified a family of interferon-inducible transmembrane (IFITM) proteins that restrict the late stage of the endocytic pathway of many enveloped viruses [49,50]. Further investigation revealed that IFITMs directly block the entry of viruses by disrupting intracellular cholesterol homeostasis [51], restricting viral membrane fusion [52], or interrupting the formation of viral fusion pores [53]. Meanwhile, the human collectins, such as mannose binding lectin (MBL) and surfactant proteins (SPs), are able to directly block virus entry. The pre-incubation of serial dilutions of MBL with HIV cell-derived living particles dramatically neutralized HIV infection [54,55]. MBL may directly inhibit HIV entry into T cells mediated by DC-SIGN, a key attachment factor for HIV invasion, via prevention of the direct HIV/DC-SIGN interaction [56]. MBL was also reported to interact with the envelope glycoproteins of Ebola and Marburg viruses, resulting in the impairment of viral entry by blocking virus-DC-SIGN interactions [57]. SP-D binds Influenza A virus, thereby inhibiting the attachment and entry of the virus by viral aggregation [58,59,60,61]. Here, our investigation shows that arthropods have evolved a similar machinery to recognize and interrupt viral entry, indicating the common ancestry of the innate immune arm presents in both mammals and arthropods.

Extracellular viruses are easily exposed to and destroyed by host immune effectors, such as antibodies in humans and antimicrobial peptides (AMPs) in insects. AMPs, regulated by NF-KappaB (REL)-mediated signaling, can directly electrostatically or hydrophobically associate with the viral surface components [62], subsequently resulting in viral inactivation [11,63,64,65]. Release of AMPs is a potent antiviral response to protect insect cells against viral infection [11,40,63,66]. For intrathoracic microinjection, the wound could elevating antimicrobial peptides (AMPs) gene expression [67]. However, in this study, the *AMPs* expression did not differ between the AaHig antibody-inoculated and pre-immune antibody-inoculated mosquitoes (S16A–S16C Fig). AaHig is capable of tethering flaviviral particles onto the cell membrane and blocking viral entry into mosquito cells. The virions exposed outside mosquito cells have a greater chance to be eliminated by AMPs and other antiviral effectors. Therefore, the direct blockade of flaviviral entry could be an effective strategy for virus killing in mosquitoes. In addition to killing viruses and infected cells, one goal of the antiviral systems is to protect the uninfected cells from viral invasion. RNA interference (RNAi), which is deemed as the major antiviral machinery in mosquitoes, however only limits viral replication in infected cells [68,69]. The system cannot function in the extracellular milieu or prevent viral spread among mosquito cells. The AaHig-mediated antiviral mechanism may complement the intracellular antiviral machinery to efficiently control viral dissemination among mosquito cells.

Mosquitoes serve as the principal vectors for a number of flaviviruses in nature. In mosquitoes, the sophisticated antiviral systems successfully limit viral infection to a tolerable level, which protects the tissues from pathological damage by viral infection. Mosquitoes do not die of flaviviral infection in nature and in our experimental system. However, infection becomes lethal to mosquitoes when AaHig function is compromised, suggesting a critical role for AaHig in restricting virus-caused damage. Notably, the AaHig-mediated antiviral machinery is restricted to the brain, and it is dispensable in other tissues such as the salivary glands. In this regard, AaHig may potentially promote flavivirus transmission in nature by enabling mosquito survival and maintaining their normal life span. Characterizing the special antiviral mechanisms of insects may greatly extend our understanding of the sophisticated interactions between mosquito-borne viruses and their vectors and therefore may provide novel strategies for arboviral disease control in the future.

## Materials and Methods

### Mosquitoes, cells and viruses

*A*. *aegypti* (the Rockefeller strain) and *C*. *pipien pallens* (the Beijing strain) were reared in a low-temperature illuminated incubator, model 818 (Thermo Electron Corporation, Waltham, MA) at 26°C and 80% humidity according to standard rearing procedures [2,69]. *Aedes albopictus* C6/36 cells were grown at 30°C in Dulbecco’s modified Eagle's medium for DENV-2 (New Guinea C strain), SINV (MX10/YN87448 strain) and JEV (SA-14 strain) production. *A*. *aegypti* Aag2 and *Drosophila* S2 cells were cultured at 28°C in Schneider’s *Drosophil*a medium for viral infection. All media were supplemented with 10% heat-inactivated fetal bovine serum, 1% L-glutamine, and 100 U/mL each of penicillin and streptomycin. The viruses were stocked in a -80°C ultra-freezer. DENV-2, SINV and JEV were titrated by both plaque formation assay (pfu) and 50% mosquito infectious dose (M.I.D._50_) as described previously [11,70,71].

### Transfection of Aag2 cells

The transfection efficiency was largely determined by the status of the Aag2 cells. The monolayer cells without aggregation were suitable for transfection. Briefly, the Aag2 cells were seeded at 3×10^6^ cells/ml per well in a 6-well plate. The cells formed a monolayer after 12 hrs of culture. Then, 0.4 μg of plasmid was premixed with Effectene (Qiagen, Cat. No# 301425) according to the manufacturer’s instructions, and consequently added to the cells. After 6–18 hrs of transfection, the medium was replaced with fresh medium. The cells were cultured for the following investigation.

### Bioinformatics

The sequences of the *Hig* genes in insects were obtained from the NCBI database. The unrooted phylogenetic tree was built with the Neighbor-joining method [72] using MEGA software v. 6.06 based on the alignment of the sequences determined using CLUSTAL W [73]. The bootstrap consensus tree was inferred from 5000 replicates. The functional modules of AaHig and DmHig were predicted using the SMART (http://smart.embl-heidelberg.de/smart/set_mode.cgi?GENOMIC=1) and Pfam (http://pfam.sanger.ac.uk/) websites. The sequence accession numbers of the Hig proteins are: *Drosophila melanogaster* Hig, NP_724772.1; *Drosophila ananassae* Hig, XP_001959877.1; *Drosophila pseudoobscura* Hig, XP_001361057.2; *Drosophila mojavensis* Hig, XP_002005215.1; *Drosophila grimshawi* Hig, XP_001987337.1; *Ceratitis capitata* Hig, XP_004526452.1; *Musca domestica* Hig, XP_005176426.1; *Anopheles gambiae* Hig, XP_001237862.2; *Aedes aegypti* Hig, AIS74715; *Culex quinquefasciatus* Hig, XP_001850783.1; *Tribolium castaneum* Hig, XP_971621.2; *Dendroctonus ponderosae* Hig, ERL89483.1; *Nasonia vitripennis* Hig, XP_001604226.2; *Cerapachys biroi* Hig, EZA58999.1; *Camponotus floridanus* Hig, EFN61193.1; *Acromyrmex echinatior* Hig, EGI66877.1; *Solenopsis invicta* Hig, EFZ22069.1; *Megachile rotundata* Hig, XP_003708514.1; *Bombus terrestris* Hig, XP_003396303.1; *Bombus impatiens* Hig, XP_003484650.1; *Apis florea* Hig, XP_003693489.1; *Apis dorsata* Hig, XP_006615530.1; *Danaus plexippus* Hig, EHJ65552.1; *Bombyx mori* Hig, NP_001108468.1.

### Antibodies and antisera generation

A flaviviral E protein 4G2 monoclonal antibody was produced from a hybridoma cell line (ATCC, Cat. No# D1-4G2-4-15). The cleaved-caspase-3 (Asp175) antibody was purchased from Cell Signaling Technology (Cat. No# 9661) [31]. The anti-horseradish peroxidase (anti-HRP) antibody was bought from Thermo Fisher Scientific (Cat. No# PA1-26409) [20,21]. The *Drosophila* Rab5 antibody was purchased from Abcam (Cat. No# ab31261), which can be used in detection of mosquito Rab5 (S21 Fig). The Wheat germ agglutinin (WGA) conjugated with Alexa Fluor-488 was used as the plasma membrane marker (Cat. No# W11261, Invitrogen) for immunofluorescence staining [74,75]. The antibodies for the tags were purchased from Medical & Biological Laboratory (MBL, Japan) and Cell Signaling Technology. For antibody generation, the *AaHig* gene was amplified from *A*. *aegypti* cDNA and cloned into a pET-28a (+) expression vector (Novagen, Cat. No# 69864–3). The cloning primers are presented in the S1 Table. The recombinant AaHig protein was expressed in the *Escherichia coli* BL21 DE3 strain, with the insoluble form in inclusion bodies. The protein was then resolved by 8 M urea and purified using a purification kit (Clontech, Cat. No# 635515). The polyclonal antibody was produced by 3 boosting immunizations of AaHig in B6 mice.

### Immuno-blockade, gene silencing and viral infection in mosquitoes

We have described the detailed procedures for gene silencing and viral challenge in mosquitoes elsewhere [2]. Briefly, female mosquitoes were cold-anesthetized on a cold tray, and subsequently 1 μg/300 nL of double-strand RNA (dsRNA) was microinjected into the mosquito thoraxes. The injected mosquitoes were allowed to recover for 3 days under standard rearing condition. The mosquitoes were then thoracically microinjected again with 10 M.I.D._50_ /300 nL (for functional investigation) or 1000 M.I.D._50_ /300 nL viruses (for the detection of gene expression) for additional investigations. In the immuno-blocking assay, we premixed the serial dilutions of the AaHig antibody with 10 M.I.D._50_ DENV-2 or JEV, and consequently microinjected the mixture into mosquitoes. The inoculated mosquitoes were reared in double containers under standard condition. At 3 and 6 days post-infection, the inoculated mosquitoes were killed and the total RNA of the whole bodies or heads was isolated to assess the viral burden with qPCR. The primers for viral detection are shown in the S1 Table.

### Detection of viral burden by qPCR

The whole bodies or heads of the infected mosquitoes were homogenized in Buffer I of an RNeasy Mini Kit (Qiagen, Cat. No# 74106) with a Pestle Grinder System (Fisher Scientific, Cat. No# 03-392-106). The detailed procedure of total RNA isolation is described in the RNeasy Kit manual. Complementary DNA (cDNA) was randomly reverse-transcribed using an iScript cDNA Synthesis Kit (Bio-Rad, Cat. No# 1708891). The viral burden was then quantified with qPCR. The primers and probes are shown in the S1 Table. The amount of virus was normalized with *A*. *aegypti* actin (*AAEL011197*). For detection in Aag2 cells, the total RNA of cultured cells was isolated for reverse transcription into cDNA. The viral burden was then measured with qPCR with the described primers and probes in the S1 Table.

### Protein generation in a *Drosophila* expression system

The gene of *AaHig* was amplified and inserted into the pMT/BiP/V5-His A vector (Invitrogen), and then the recombinant plasmids were transfected into *Drosophila* S2 cells in combination with a Hygromycin selection vector pCoHygro for stable cell construction. The primers for PCR and gene cloning are shown in the S1 Table. The stable cell screening and purification were described in our previous study [2,3]. Briefly, the transfected cells were selected using 300 μg/mL Hygromycin-B (Invitrogen, Cat. No# 10687–010) for 4 weeks. The resistant cells were grown in spinner flasks, switched to Express Five serum-free medium (GIBCO, Invitrogen, Cat. No# 10486025) for 3 days, and induced with copper sulfate at a final concentration of 500 μM for 4 days. The culture medium was collected for protein purification with a metal affinity resin (Clontech, Cat. No# PT1320-1). The protein was eluted with 150 mM imidazole, extensively dialyzed against PBS (pH 7.8), and concentrated via centrifugal filtration through a 5-kDa filter (Millipore Corp., Cat. No# pLCC07610). The protein concentration was measured using a Protein Assay Dye (Bio-Rad, Cat. No#500–0006) and a Nanodrop 2000c spectrophotometer (Thermo Scientific). The protein purity was checked with sodium dodecyl sulfate polyacrylamide gel electrophoresis, and the specificity of purification was confirmed by western blotting.

### Co-immunoprecipitation (co-IP)

For the assay with purified proteins, five micrograms each of purified DENV-2 E and AaHig were incubated at 4°C for 2 hrs. Subsequently, 1 μg of baited antibody was added to pull down the protein complex. For the experiment with cell supernatant/lysate, the recombinant plasmids were transiently transfected into *Drosophila* S2 cells with an Effectene transfection kit (Qiagen, Cat. No# 301425). The cell supernatant/lysate was incubated with purified protein overnight at 4°C for the pull-down assay. To investigate the interaction between AaHig and DENV virions in the infected cells, pAc-AaHig was transfected in mosquito Aag2 cells, and subsequently the cells were infected by 5 M.O.I. DENV-2 at 12 hrs post transfection. The uninfected cells transfected by pAc-AaHig were used as a mock control. After 48 hrs infection, the cells were lysated and an anti-flaviviral E 4G2 mAb was added into the lysate for the pull-down assay. The experimental details are described in the Pierce Classic IP kit product manual (Thermo Scientific, Cat. No# 26146).

### ELISA

The microtiter plate (Nunc, Roskilde, Denmark) was coated with 2 μg purified protein overnight at 4°C. After 5 washes with PBS containing 0.05% Tween 20 (PBST), the supernatant/lysate of transfected cells was added to each well and incubated at room temperature (RT) for 2 hrs. The wells were then washed 5 times with PBST. Primary antibody was added, and incubation continued at RT for 2 hrs. The wells were washed again, and 100 μL of secondary IgG-horseradish peroxidase was added. After incubation at RT for 1 hr, a commercial peroxidase substrate system was used (Kirkegaard & Perry Laboratories, Inc., MA, Cat. No# 50-76-11), and the optical density at 450 nm was measured with an ELISA reader.

The interaction between AaHig and DENV-2 virions was also measured by ELISA. In the procedure, the plate was coated with 2 μg of purified AaHig protein at 4°C overnight. After 5 washes with PBST, 2 μg of purified inactivated DENV-2 virions (MicroBix, Canada, Cat. No# EL-22-02-001) in PBS was added to each well and incubated for 2 hrs at 4°C. After washing with PBST, a flavivirus E protein 4G2 mAb was added, and further incubated for 2 hrs at 4°C. The analysis followed the procedure is outlined above.

### Viral attachment and entry assays

We assessed the viral attachment and internalization by Aag2 and C6/36 cells. For the attachment assay, the purified AaHig protein was premixed with viruses, and the cells were consequently pre-adsorbed with the mixture at 4°C for a time course. After five washes with pre-cooled PBS buffer, the cells were collected to isolate total RNA. The copy number of the viral genome was determined by qPCR. For the entry assay, AaHig and the viruses were premixed, and then the cells were incubated with the mixture at 28°C (Aag2) / 30°C (C6/36) for a time course. After washing with PBS at RT, the cells were used to determine the viral burden. For the assay at 48 hrs, the cells were washed 5 times after a 1 hr incubation, then cultured at 28°C (Aag2) / 30°C (C6/36) for an additional 48 hrs. The cells were collected to isolate the total RNA for viral genomic detection by qPCR.

### Imaging of mosquito brains

The protocol of *A*. *aegypti* brain isolation and staining was described in previous studies [21,76]. Briefly, the mosquito heads were cut off, then fixed in 4% paraformaldehyde at 4°C for 1 week. The brains were dissected by fine forceps and probes, treated with 2% Triton X-100 for 1 hr, and then blocked by the BD Perm/Wash buffer (BD, Cat. No# 51-2091KZ). The tissues were placed on sialylated slides (PGC Scientific, USA). After stained by primary and secondary antibodies, the tissues were imaged using a Zeiss LSM 780 meta confocal microscope (Carl Zeiss, Germany) with a Multi-Track mode.

### *In situ* detection of apoptotic cell death

The apoptosis of mosquito tissues was determined by a terminal deoxynucleotidyl transferase (TdT)-mediated dUTP nick end-labeling (TUNEL) assay. After six days post DENV-2 infection with or without AaHig antibody treatment, the mosquito heads were cut off and fixed in 4% paraformaldehyde for brain isolation. The TUNEL assay was performed by a cell death *in situ* detection kit, Fluorescein (Roche, Cat. No# 11684817910). A FITC filter was used to detect TUNEL staining (green color). TUNEL-positive staining patterns were acquired by a Zeiss LSM 780 meta confocal microscope (Carl Zeiss, Germany) with a Multi-Track mode.

### Structured illumination microscopy imaging

Aag2 cells were seeded in a glass bottle cell culture dish (Nest Biotechnology, Cat. No# 801001) for 12 hrs. The 2 μg purified AaHig protein was premixed with 10 M.O.I. DENV-2 in 500 μL medium, and the mixture was then added to the cells. After the cells were cultured at 28°C for a time course, the cells were washed with PBS and fixed in 4% paraformaldehyde for 1 hr. Permeabilization was performed using 0.05% Triton X-100 for 30 min. After three washes in PBS, the cells were blocked using the Perm/Wash buffer (BD, Cat. No# 51-2091KZ). After the primary and secondary antibodies staining, the cells were imaged using a Zeiss ELYRA PS.1 structured illumination microscope (Carl Zeiss, Germany).

### Detection of phenoloxidase (PO) activity

After 48 hrs of transfection, the Aag2 cells were stimulated by 1 M.O.I. DENV-2 or JEV for 30 min. The supernatant was collected by centrifugation at 3000 rpm for 10 min at 4°C in order to remove debris. The PO activity assays were performed in 96-well plates. One hundred microliters of 50 mM Sodium Phosphate buffer (pH 6.5) containing 2 mM dopamine (the substrate for the PO, Sigma, Cat. No# D-9628) was added to 20 μL of cell culture medium. PO activity was monitored over 30 min by measuring the absorbance at 490 nm using a plate reader [77,78].

### H_2_O_2_ assay

After 6 hrs of infection, the mosquito parts including heads, carcasses and whole bodies, were collected into the PBS buffer with 2 mg/ml of the catalase inhibitor 3-amino-1,2,4-triazole (Sigma, Cat. No# A8056-10G). After homogenization, the samples were filtered through a spin filter with a 10K molecular weight cutoff (Corning Spin-XUF, Corning, Cat. No# 431486). The elution from each experimental group was then collected and tested using a hydrogen peroxide assay kit (BioVision, Cat. No# K265-200). The fluorescence intensity was detected at Excitation/Emission = 550/590 using a fluorescence microplate reader, according to the manufacturer's instructions. The value was normalized by the total amount of proteins in the sample, as determined by a Protein Assay Dye (Bio-Rad, Cat. No# 500–0006) and a Nanodrop 2000c spectrophotometer (Thermo Scientific).

### Particles uptake assay

The nano-beads with a 10–100 nm diameter (Cat. No# LB1-1ML, Sigma), which mimic the size of the viruses, was selected to perform a particle uptake assay in mosquito Aag2 cells. The beads were pre-labeled by Fluorescein isothiocyanat (FITC). AaHig protein was premixed with the beads, then incubated the materials with Aag2 cells at 28°C for 30 min. The same amount of BSA mixed with the beads was used as the mock control. The incubated cells were washed 3 times by PBS buffer, and then treated by 0.2%Trypan Blue to quench the fluorescence of the beads attached on the cell surface [35,79]. The amount of uptake beads, which had been internalized into mosquito cells, was measured by the flow cytometry. The treated cells were then examined using a FACS Calibur flow cytometer (BD Biosciences, San Diego, CA). Dead cells were excluded on the basis of forward and side light scatter. Data were analysed using FlowJo software.

## Supporting Information

S1 Fig Generation of murine AaHig polyclonal antibody. (A) Purification of AaHig recombinant protein in *E*. *coli*. The *AaHig* fragment (1bp-2436bp) was cloned into pET-28a (+) DNA vector and expressed in *E*. *coli* BL21 DE3 strain. The recombinant protein, expressed in inclusion body, was dissolved in 8M Urea and purified by Ni-His column for antibody generation. (B) Validation of AaHig polyclonal antibody. A murine AaHig polyclonal antibody was used to probe *E*. *coli*- or S2-expressed AaHig recombinant protein. The same samples probed by murine pre-immune antibody served as a negative control.
(PDF)

S2 Fig The distribution of inoculated AaHig antibody in mosquito tissues. The 1:10 diluted AaHig murine antibody was microinjected into mosquito thorax and the various tissues were isolated for staining by anti-mouse IgG-Alexa 546. Nuclei were stained with To-Pro-3 iodide (Blue). Images were examined using a Zeiss LSM 780 meta confocal. Ten mosquitoes have been dissected for analysis with the similar results.
(PDF)

S3 Fig *AaHig* regulation in various mosquito tissues by DENV-2 infection. DENV-2 (1000 M.I.D._50_) or PBS was microinjected into mosquitoes. Total RNA was isolated from whole mosquitoes, heads, salivary glands, midguts, hemolymph and carcasses at 3 (A), 6 (B), 9 (C), 12 (D) days post DENV-2 infection to determine *AaHig* expression by SYBR Green qPCR. The qPCR primers of *AaHig* were described in the S1 Table. The data were represented as the mean ± standard error.
(PDF)

S4 Fig AaHig coats on the membrane of Aag2 cells by Z-Stack imaging. The pAc-AaHig recombinant plasmid was transfected into Aag2 cells. AaHig was stained by anti-V5 antibody and anti-mouse IgG Alexa-546 (Red). The plasma membrane was stained by the Wheat Germ Agglutinin (WGA) conjugated with Alexa-488 (Green). Nuclei were stained with To-Pro-3 iodide (Blue). Images were examined using a Zeiss LSM 780 meta confocal microscope with a Z-stack model.
(PDF)

S5 Fig The subcellular localization of AaHig. The subcellular fractionations, including nucleus, mitochondria, cytoplasm and plasma membrane, were separated and validated by their featured markers. AaSR-C, a transmembrane protein in *A*. *aegypti*, was used as a marker of plasma membrane [11].
(PDF)

S6 Fig The effect of dsRNA-mediated *AaHig* silencing in DENV-2 infection of *A. aegypti*. (A-C) Inoculation of *AaHig* dsRNA significantly decreased the *AaHig* expression in whole mosquito bodies and heads at both the mRNA (A and B) and protein (C) levels. The *AaHig* abundance was assessed by SYBR Green qPCR (A and B) and western blotting with an AaHig antibody (C) at 6 days post microinjection in *A*. *aegypti*. (D-E) Silencing *AaHig* enhanced DENV-2 infection in *A*. *aegypti*. 10 M.I.D._50_ DENV-2 were inoculated at 3 days post *AaHig* dsRNA inoculation. The viral load of whole bodies (D) and heads (E) was assessed at 3 days (i) and 6 days (ii) post-infection by Taqman qPCR and normalized with *A*. *aegypti actin* (*AAEL011197*). The primers and probes of qPCR were described in the S1 Table. The experiment was repeated two times with similar results. One dot represents 1 mosquito and the horizontal line represents the median value. The data were statistically analyzed by the non-parametric *Mann-Whitney* test.
(PDF)

S7 Fig The distribution of AaHig antibody in the *A. aegypti* brain. The 10-fold diluted murine AaHig antibody (Ab) was microinjected into the thorax of mosquitoes. At serial time points, the mosquito brains were fixed and dissected for staining by anti-mouse IgG Alexa-546 (Red). Nuclei were stained blue with To-Pro-3 iodide. Images were examined using the 10× (A) and 63× (B) objective lens of a Zeiss LSM 780 meta confocal.
(PDF)

S8 Fig Comparison of the distribution of murine AaHig antibody in different mosquito brains. The 1:10 diluted AaHig murine antibody was microinjected into mosquito thorax and the brain tissues were isolated for staining by anti-mouse IgG-Alexa 546. Nuclei were stained with To-Pro-3 iodide (Blue). Images were examined using a Zeiss LSM 780 meta confocal microscope.
(PDF)

S9 Fig Immuno-blockade of AaHig significantly enhanced the DENV replication in the mosquitoes infected by oral feeding. The mosquitoes were fed with the Vero cells-generated DENV-2 and fresh human blood via Hemotek oral feeding system. Subsequently, the anti-AaHig antibody was intrathoracically microinjected into the mosquitoes 3 days after viral blood feeding. The fed mosquitoes that were inoculated by pre-immune antibody were used as a negative control. The mosquitoes were reared under standard condition. After removing the uninfected mosquitoes, the DENV load in mosquito bodies (A and B) and heads (C and D) was measured via qPCR at 6 days and 9 days after the oral infection. The primers and probes of qPCR were described in the S1 Table. The results were pooled from two parallel experiments. One dot represents 1 mosquito and the horizontal line represents the median value. The data were statistically analyzed by the non-parametric *Mann-Whitney* test.
(PDF)

S10 Fig Detection of viral burden in salivary glands and midguts of *A. aegypti*. The murine AaHig antibody with 10-fold dilutions was premixed with 10 M.I.D._50_ DENV-2 to co-microinject into the mosquitoes. The salivary glands (A) and midguts (B) were then dissected to examine the viral load at 3 (i) and 6 (ii) days post-infection via TaqMan qPCR and normalized against *A*. *aegypti actin*. The results were combined from 2 independent experiments. The data were analyzed statistically using the non-parametric *Mann-Whitney* test.
(PDF)

S11 Fig *AaHig* silencing did not influence the SINV infection in *A*. *aegypti*. (A-D) The effect of *AaHig* silencing in SINV infection of *A*. *aegypti*. 10 M.I.D._50_ SINV were inoculated at 3 days post *AaHig* dsRNA inoculation. The viral load of whole bodies (A and B) and heads (C and D) was assessed 3 days and 6 days post-infection via qPCR and normalized with *A*. *aegypti actin*. The qPCR primers and probes were described in the S1 Table. The experiment was repeated two times with similar results. One dot represents 1 mosquito and the horizontal line represents the median value. The data were statistically analyzed by non-parametric *Mann-Whitney* test. (E) The expression of Sindbis Envelope proteins in *Drosophila* S2 cells. Three Sindbis *Envelope* genes (E1, E2 and E3) with FLAG tag were cloned into the pMT/Bip/V5-His A vector and expressed in the S2 cell supernatant. The supernatant from empty vector-transfected S2 cells was used as a mock. The E proteins were detected with an anti-FLAG antibody via western blotting. (F) The Sindbis E proteins do not interact with AaHig by a co-IP assay. Three Sindbis *Envelope* genes (E1, E2 and E3) were cloned into the pMT/Bip/V5-His A vector, and subsequently co-expressed with AaHig in the S2 cell supernatant. The protein complex was pulled down with an anti-FLAG antibody and detected using an anti-V5-HRP antibody. We reproduced the experiment 2 times.
(PDF)

S12 Fig The interaction between AaHig and JEV E protein. The interaction of purified AaHig (V5) and JEV E (FLAG) proteins was determined by ELISA (A) and co-IP (B). In the ELISA detection, the binding was probed by mouse anti-FLAG-HRP antibody. The data were presented as the mean ± standard error. The experiment was reproduced 3 times. In the co-IP assay, the protein complex was pulled down with an anti-V5 antibody and detected using a mouse anti-FLAG-HRP antibody. We reproduced the experiments 3 times.
(PDF)

S13 Fig The membrane-bound capability of AaHig truncations on mosquito Aag2 cells. The seven truncations, in which the functional domains of AaHig were sequentially deleted, were expressed in mosquito Aag2 cells. The membrane location of these truncations was determined by immunofluorescence staining. The AaHig truncations were stained with an anti-V5 antibody (Red); the cellular membrane was stained by a plasma membrane marker, Wheat Germ Agglutinin (WGA) (Green); nuclei were stained blue with To-Pro-3 iodide (Blue). Images were examined using a Zeiss LSM 780 meta confocal 63×objective lens.
(PDF)

S14 Fig The antiviral effect of Hig protein in mammalian cells and insect cells. (A-B) AaHig did not reduce the DENV infection in the mammalian cells. 2μg AaHig purified protein and 0.01 M.O.I. DENV-2 was incubated with human A549 (A) and Vero (B) cells. The same amount of BSA with DENV-2 was used as negative controls. After 48 hours, the viral load was determined by Taqman qPCR and normalized by human *GAPDH*. The data were presented as the mean ± standard error. The results were combined from 2 independent experiments. (C) The ectopic expression of *Drosophila melanogaster Hig* (*DmHig*) in S2 cells. The gene of *DmHi*g were cloned into the pAc5.1/V5-His A vector and expressed in the S2 cell supernatant. The supernatant from empty vector-transfected S2 cells was used as a mock. The recombinant protein was detected with an anti-V5 antibody via western blotting. (D-E) The antiviral effect of *DmHig* in insect cells. DmHig was overexpressed in mosquito Aag2 (D) and *Drosophila* S2 (E) cells. After 48 hrs, the 0.01 M.O.I. DENV-2 was added into the cells, and subsequently the viral load was determined by Taqman qPCR and normalized by *A*. *aegypti* and *D*. *melanogaster actins*. The data were presented as the mean ± standard error. The results were combined from 3 independent experiments.
(PDF)

S15 Fig AaHig cannot coat on the membrane of Vero cells by an immuno-staining assay. The 5 ug purified AaHig protein was incubated with the Vero cells for 1 hr. After 5 washing by PBS buffer, the cells were fixed by 4% PFA and staining by AaHig antibody. The WGA conjugated with Alexa Fluor-488 was used to stain the cellular membrane. The Aag2 cells with the same amount of AaHig served as a positive control.
(PDF)

S16 Fig Immuno-blockade of AaHig does not alter the expression of immune-related genes. The murine AaHig antibody with 10-fold dilution was microinjected in *A*. *aegypti*. The same amount of diluted pre-immune antibody was used as a negative control. The expression of immune-related genes, such as *AMPs* (A-C), *A*. *aegypti Ago2* (*AaAgo2*) (D), *A*. *aegypti Dicer2* (*AaDicer 2*) (E), *A*. *aegypti Duox1* (*AaDuox1*) (F) and *A*. *aegypti Duox2* (*AaDuox2*) (G), was determined by qPCR at 6 hrs post-inoculation in mosquitoes. The amount of genes was normalized by *A*. *aegypti actin*.
(PDF)

S17 Fig The role of AaHig in the activation of melanization and reactive oxygen systems. (A-B) Overexpression of AaHig did not regulate the melanization activity in Aag2 cells. The phenoloxidase (PO) activity was measured in DENV-2 (A) or JEV (B) infected or mock cells. The regulation of PO activity was presented by the fold change than that in uninfected pAc-GFP-transfected mock cells. (C) Knockdown of *AaHig* did not influence the H_2_O_2_ release in various mosquito parts. The dsRNA mediated silencing was performed in mosquitoes. The same amount of *GFP* dsRNA was inoculated into mosquito thorax as a negative control. At 3 days later, 1000 M.I.D._50_ DENV-2 were microinjected into mosquitoes. The various mosquito parts were collected at 6 hrs post infection. The concentration of H_2_O_2_ was measured by a Hydrogen Peroxide Assay Kit. The result was presented by the fold change calculated by H_2_O_2_ concentration in the infected mosquitoes / that in uninfected mosquitoes.
(PDF)

S18 Fig AaHig reduces JEV entry in mosquito cells. (A-B) JEV attachment assay at 4°C. The serial concentration of purified AaHig protein was premixed with 5 M.O.I. JEV on ice, and then the Aag2 (A) and C6/36 (B) cells were incubated with the mixture for a time course at 4°C. The cells were washed 5 times by cold PBS buffer and collected at certain time points for total RNA isolation. (C-D) JEV internalization assay. The serial concentration of purified AaHig protein was premixed with 5 M.O.I. JEV, and consequently the mixture was incubated into the Aag2 cells at 28°C (C) and C6/36 cells at 30°C (D). The cells were washed 5 times by PBS at room temperature and collected for detection. (A-D) For the assay at 48 hrs, the cells were washed 5 times after 1 hr incubation at 4°C (A and B) or 28°C/30°C (C and D), and consequently cultured at 28°C or 30°C for an additional 48 hrs. The viral genome was determined by Taqman qPCR and normalized by *A*. *aegypti actin*. The data were presented as the mean ± standard error. The results were combined from 3 independent experiments. *, *p<0*.*05*; **, *p<0*.*01*; ***, *p<0*.*001*.
(PDF)

S19 Fig AaHig does not generally sequester unrelated particles on plasma membrane. The nano-beads with a 10–100 nm diameter, which mimic the size of viruses, were selected to perform a particle uptake assay in mosquito Aag2 cells. The beads were pre-labeled by Fluorescein isothiocyanat (FITC). AaHig protein was premixed with the beads, then incubated the mixture with Aag2 cells at 28°C for 30 min. The same amount of BSA mixed with the beads was used as a negative control. The mock group was the cells without the beads. After the incubation, the cells were washed 3 times by PBS buffer, and then treated by 0.2%Trypan Blue to quench the fluorescence of the beads attached on the cell surface. The amount of uptake beads, which had been internalized into mosquito cells, was measured by the flow cytometry. We reproduced these experiments 3 times.
(PDF)

S20 Fig Over-expression of *AaHig* does not influence the Sindbis burden in the human and mosquito cells. The 2 μg AaHig protein was premixed with 5 M.O.I. Sindbis virus (SINV), and then we incubated the materials with human A549 cells (A) at 37°C or mosquito Aag2 cells (B) at 28°C for a serial time course. The same amount of BSA mixed with viruses was used as a mock control. The SINV load was determined by qPCR and normalized by human or mosquito *actins*. The experiment was repeated by three times with the similar results.
(PDF)

S21 Fig Validation of *Drosophila* Rab5 polyclonal antibody. A rabbit anti-*Drosophila* Rab5 polyclonal antibody can efficiently probe Rab5 proteins in the lysates of S2 and Aag2 cells.
(PDF)

S1 Table Primers and probes for qPCR, dsRNA synthesis and genes cloning. (PDF)
