# Supplementary material for: A Neuron-Specific Antiviral Mechanism Prevents Lethal Flaviviral Infection of Mosquitoes
Source: PLoS Pathog. 2015 Apr 27;11(4):e1004848. doi: 10.1371/journal.ppat.1004848 (PMC4411065; doi:10.1371/journal.ppat.1004848)
Supplement: S1 Fig — (A) Purification of AaHig recombinant protein in E. coli. The AaHig fragment (1bp-2436bp) was cloned into pET-28a (+) DNA vector and expressed in E. coli BL21 DE3 strain. The recombinant protein, expressed in inclusion body, was dissolved in 8M Urea and purified by Ni-His column for antibody generation. (B) Validation of AaHig polyclonal antibody. A murine AaHig polyclonal antibody was used to probe E. coli- or S2-expressed AaHig recombinant protein. The same samples probed by murine pre-immune antibody served as a negative control. (PDF) [file ppat.1004848.s001.pdf]

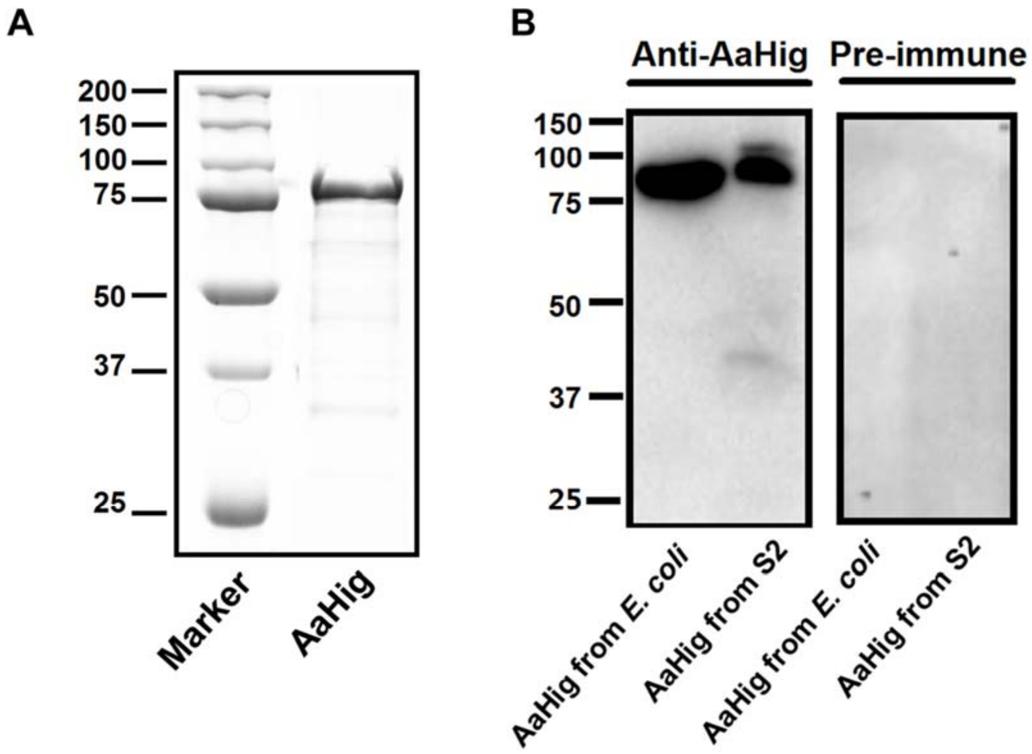

S1 Fig. Generation of murine AaHig polyclonal antibody

(A) Purification of AaHig recombinant protein in *E. coli*. The *AaHig* fragment (1bp-2436bp) was cloned into pET-28a (+) DNA vector and expressed in *E. coli* BL21 DE3 strain. The recombinant protein, expressed in inclusion body, was dissolved in 8M Urea and purified by Ni-His column for antibody generation.

(B) Validation of AaHig polyclonal antibody. A murine AaHig polyclonal antibody was used to probe *E.coli*- or S2-expressed AaHig recombinant protein. The same samples probed by murine pre-immune antibody served as a negative control.
