# Supplementary material for: A Neuron-Specific Antiviral Mechanism Prevents Lethal Flaviviral Infection of Mosquitoes
Source: PLoS Pathog. 2015 Apr 27;11(4):e1004848. doi: 10.1371/journal.ppat.1004848 (PMC4411065; doi:10.1371/journal.ppat.1004848)
Supplement: S2 Fig — The 1:10 diluted AaHig murine antibody was microinjected into mosquito thorax and the various tissues were isolated for staining by anti-mouse IgG-Alexa 546. Nuclei were stained with To-Pro-3 iodide (Blue). Images were examined using a Zeiss LSM 780 meta confocal. Ten mosquitoes have been dissected for analysis with the similar results. (PDF) [file ppat.1004848.s002.pdf]

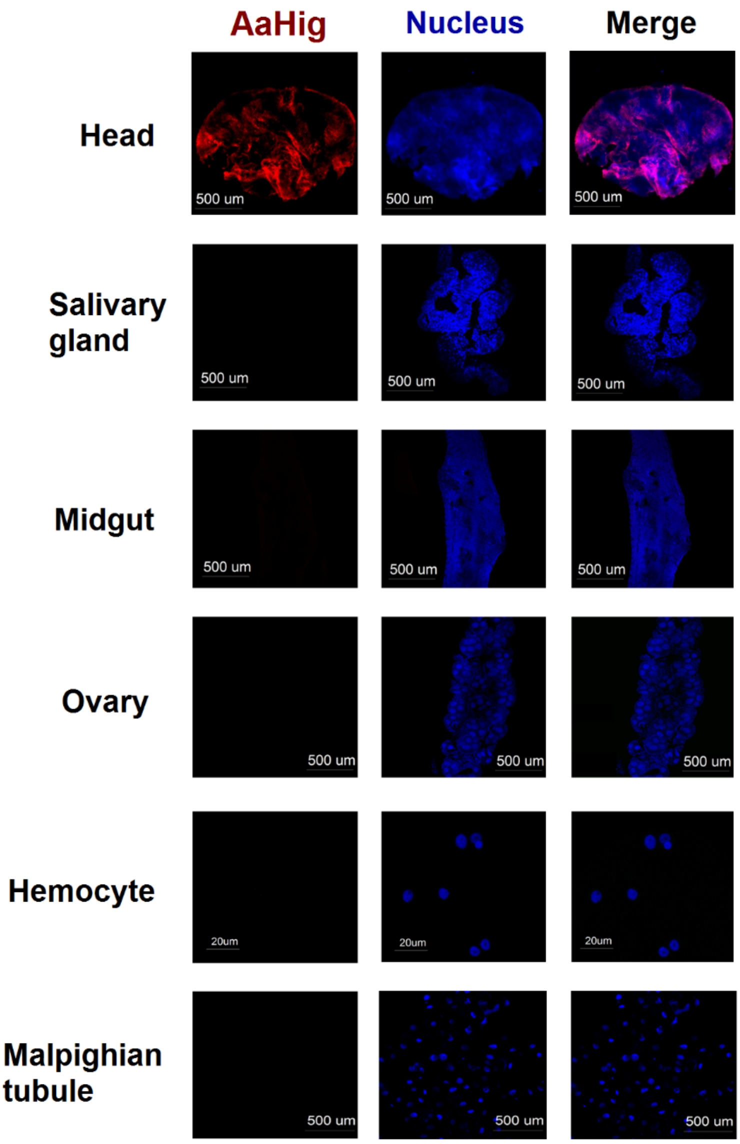

S2 Fig. The distribution of inoculated AaHig antibody in mosquito tissues

The 1:10 diluted AaHig murine antibody was microinjected into mosquito thorax and the various tissues were isolated for staining by anti-mouse IgG-Alexa 546. Nuclei were stained with To-Pro-3 iodide (Blue). Images were examined using a Zeiss LSM 780 meta confocal. Ten mosquitoes have been dissected for analysis with the similar results.
