# Supplementary material for: A Neuron-Specific Antiviral Mechanism Prevents Lethal Flaviviral Infection of Mosquitoes
Source: PLoS Pathog. 2015 Apr 27;11(4):e1004848. doi: 10.1371/journal.ppat.1004848 (PMC4411065; doi:10.1371/journal.ppat.1004848)
Supplement: S3 Fig — DENV-2 (1000 M.I.D.50) or PBS was microinjected into mosquitoes. Total RNA was isolated from whole mosquitoes, heads, salivary glands, midguts, hemolymph and carcasses at 3 (A), 6 (B), 9 (C), 12 (D) days post DENV-2 infection to determine AaHig expression by SYBR Green qPCR. The qPCR primers of AaHig were described in the S1 Table. The data were represented as the mean ± standard error. (PDF) [file ppat.1004848.s003.pdf]

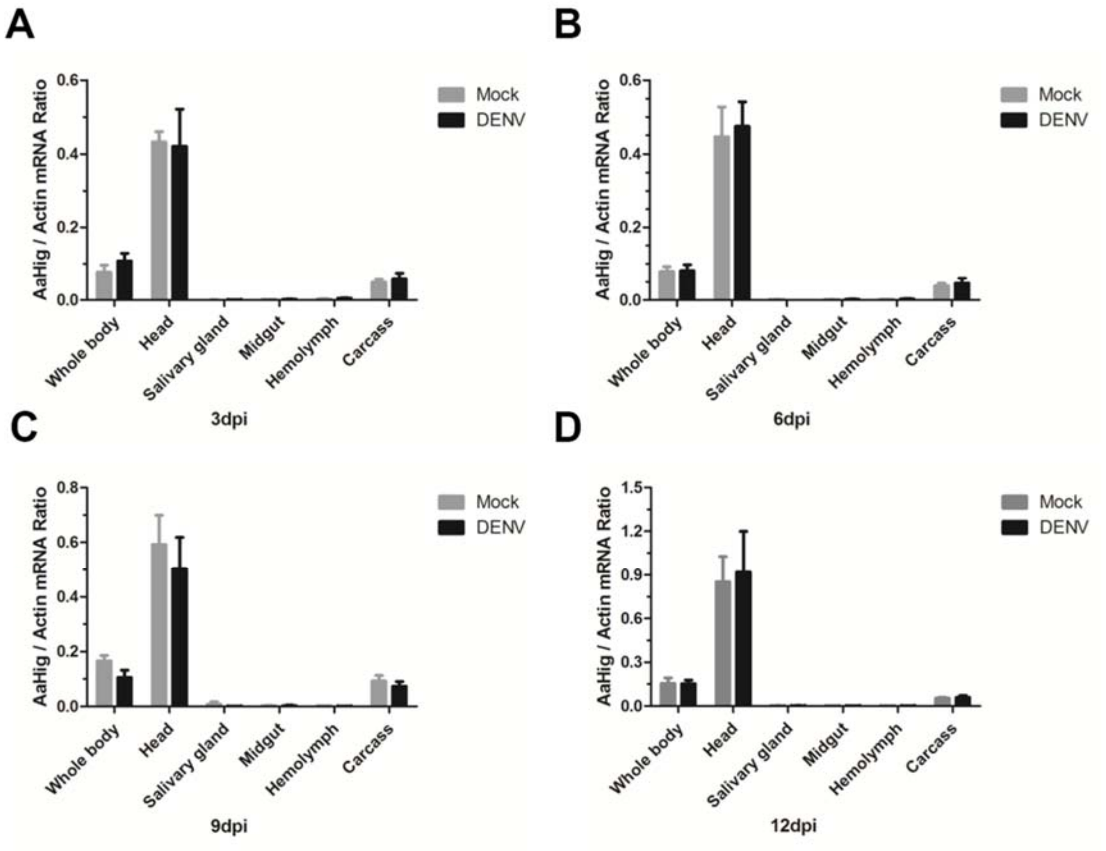

S3 Fig. *AaHig* regulation in various mosquito tissues by DENV-2 infection

DENV-2 (1000 M.I.D.$_{50}$) or PBS was microinjected into mosquitoes. Total RNA was isolated from whole mosquitoes, heads, salivary glands, midguts, hemolymph and carcasses at 3 (A), 6 (B), 9 (C), 12 (D) days post DENV-2 infection to determine *AaHig* expression by SYBR Green qPCR. The qPCR primers of *AaHig* were described in the S1 Table. The data were represented as the mean ± standard error.
