# Supplementary material for: A Neuron-Specific Antiviral Mechanism Prevents Lethal Flaviviral Infection of Mosquitoes
Source: PLoS Pathog. 2015 Apr 27;11(4):e1004848. doi: 10.1371/journal.ppat.1004848 (PMC4411065; doi:10.1371/journal.ppat.1004848)
Supplement: S4 Fig — The pAc-AaHig recombinant plasmid was transfected into Aag2 cells. AaHig was stained by anti-V5 antibody and anti-mouse IgG Alexa-546 (Red). The plasma membrane was stained by the Wheat Germ Agglutinin (WGA) conjugated with Alexa-488 (Green). Nuclei were stained with To-Pro-3 iodide (Blue). Images were examined using a Zeiss LSM 780 meta confocal microscope with a Z-stack model. (PDF) [file ppat.1004848.s004.pdf]

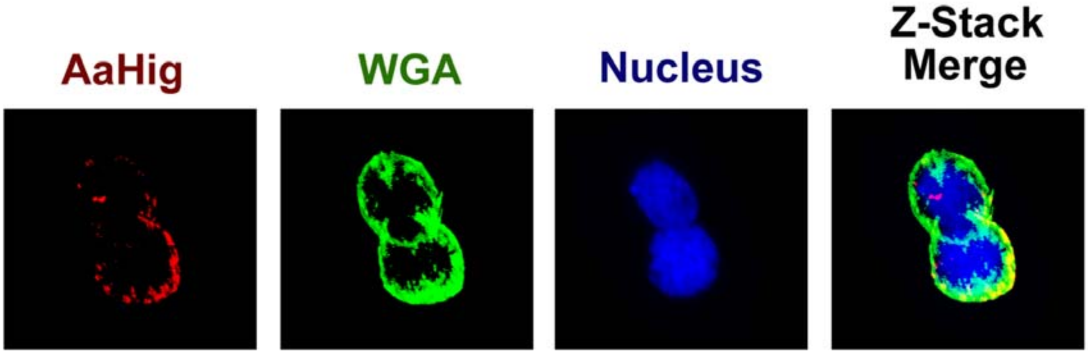

S4 Fig. AaHig coats on the membrane of Aag2 cells by Z-Stack imaging

The pAc-AaHig recombinant plasmid was transfected into Aag2 cells. AaHig was stained by anti-V5 antibody and anti-mouse IgG Alexa-546 (Red). The plasma membrane was stained by the Wheat Germ Agglutinin (WGA) conjugated with Alexa-488 (Green). Nuclei were stained with To-Pro-3 iodide (Blue). Images were examined using a Zeiss LSM 780 meta confocal microscope with a Z-stack model.
