# Supplementary material for: A Neuron-Specific Antiviral Mechanism Prevents Lethal Flaviviral Infection of Mosquitoes
Source: PLoS Pathog. 2015 Apr 27;11(4):e1004848. doi: 10.1371/journal.ppat.1004848 (PMC4411065; doi:10.1371/journal.ppat.1004848)
Supplement: S5 Fig — The subcellular fractionations, including nucleus, mitochondria, cytoplasm and plasma membrane, were separated and validated by their featured markers. AaSR-C, a transmembrane protein in A. aegypti, was used as a marker of plasma membrane [11]. (PDF) [file ppat.1004848.s005.pdf]

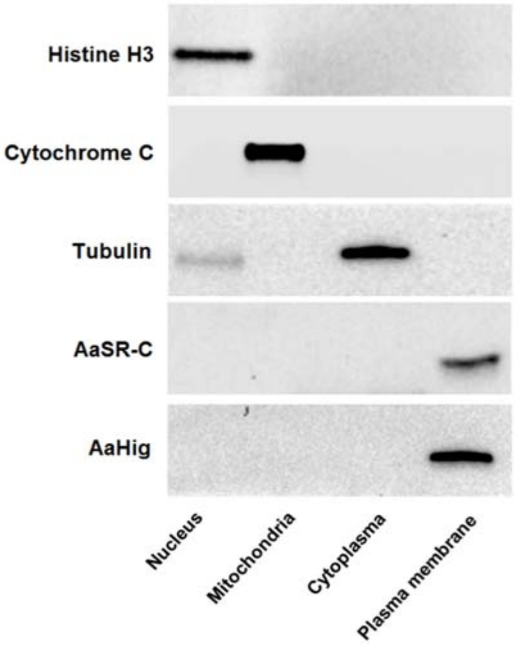

S5 Fig. The subcellular localization of AaHig

The subcellular fractionations, including nucleus, mitochondria, cytoplasm and plasma membrane, were separated and validated by their featured markers. AaSR-C, a transmembrane protein in *A. aegypti*, was used as a marker of plasma membrane [11].
