# Supplementary material for: A Neuron-Specific Antiviral Mechanism Prevents Lethal Flaviviral Infection of Mosquitoes
Source: PLoS Pathog. 2015 Apr 27;11(4):e1004848. doi: 10.1371/journal.ppat.1004848 (PMC4411065; doi:10.1371/journal.ppat.1004848)
Supplement: S6 Fig — (A-C) Inoculation of AaHig dsRNA significantly decreased the AaHig expression in whole mosquito bodies and heads at both the mRNA (A and B) and protein (C) levels. The AaHig abundance was assessed by SYBR Green qPCR (A and B) and western blotting with an AaHig antibody (C) at 6 days post microinjection in A. aegypti. (D-E) Silencing AaHig enhanced DENV-2 infection in A. aegypti. 10 M.I.D.50 DENV-2 were inoculated at 3 days post AaHig dsRNA inoculation. The viral load of whole bodies (D) and heads (E) was assessed at 3 days (i) and 6 days (ii) post-infection by Taqman qPCR and normalized with A. aegypti actin (AAEL011197). The primers and probes of qPCR were described in the S1 Table. The experiment was repeated two times with similar results. One dot represents 1 mosquito and the horizontal line represents the median value. The data were statistically analyzed by the non-parametric Mann-Whitney test. (PDF) [file ppat.1004848.s006.pdf]

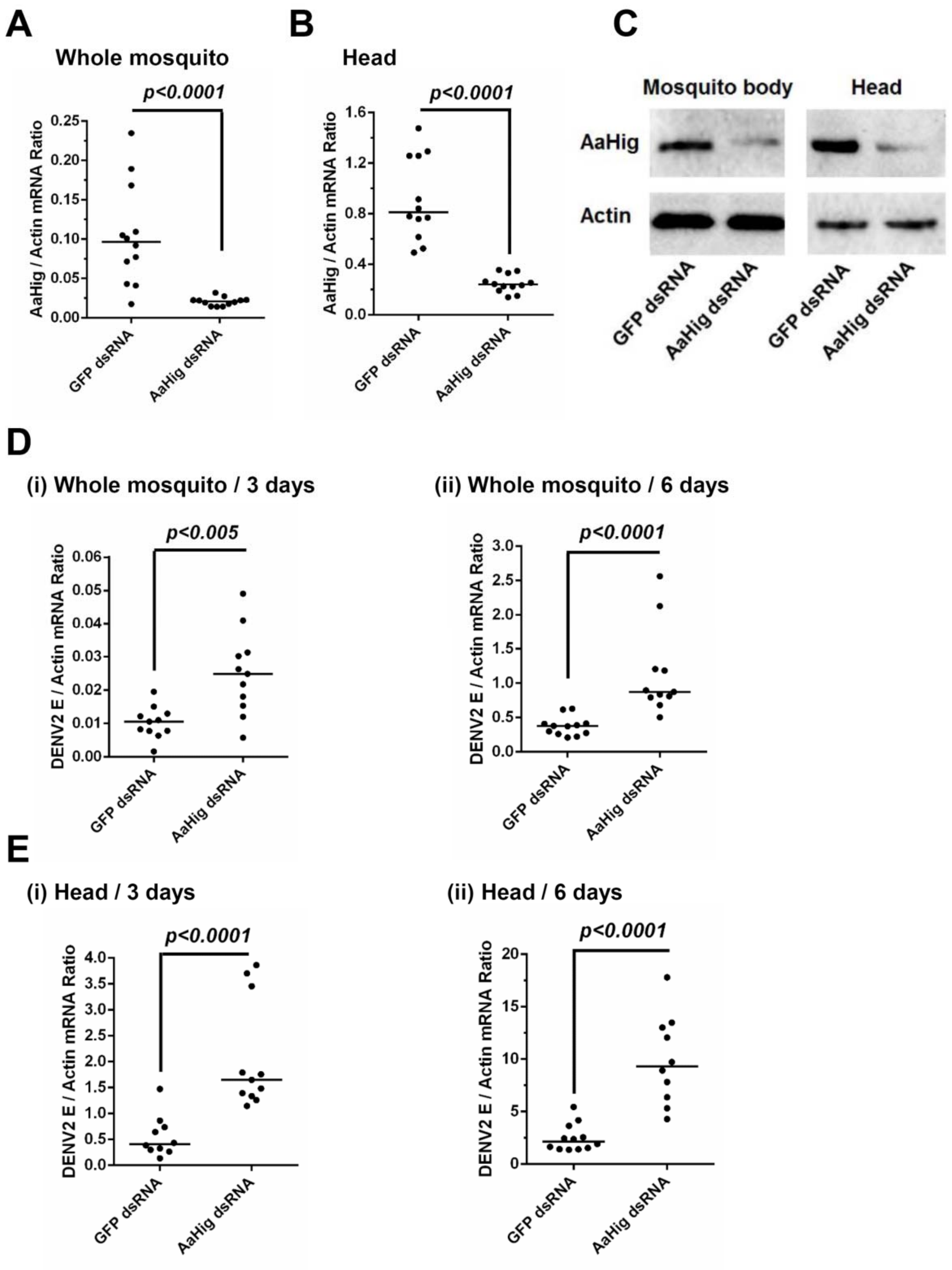

S6 Fig. The effect of dsRNA-mediated *AaHig* silencing in DENV-2 infection of *A. aegypti*

(A-C) Inoculation of *AaHig* dsRNA significantly decreased the *AaHig* expression in the whole mosquitoes and heads at both the mRNA (A and B) and protein (C) levels. The *AaHig* abundance was assessed by SYBR Green qPCR (A and B) and western blotting with an AaHig antibody (C) at 6 days post microinjection in *A. aegypti*.

(D-E) Silencing *AaHig* enhanced DENV-2 infection in *A. aegypti*. 10 M.I.D.$_{50}$ DENV-2 were inoculated at 3 days post *AaHig* dsRNA inoculation. The viral load of whole bodies (D) and heads (E) was assessed at 3 days (i) and 6 days (ii) post-infection by Taqman qPCR and normalized with *A. aegypti actin* (*AAEL011197*). The primers and probes of qPCR were described in the S1 Table. The experiment was repeated two times with similar results. One dot represents 1 mosquito and the horizontal line represents the median value. The data were statistically analyzed by the non-parametric *Mann-Whitney* test.
