# Supplementary material for: A Neuron-Specific Antiviral Mechanism Prevents Lethal Flaviviral Infection of Mosquitoes
Source: PLoS Pathog. 2015 Apr 27;11(4):e1004848. doi: 10.1371/journal.ppat.1004848 (PMC4411065; doi:10.1371/journal.ppat.1004848)
Supplement: S7 Fig — The 10-fold diluted murine AaHig antibody (Ab) was microinjected into the thorax of mosquitoes. At serial time points, the mosquito brains were fixed and dissected for staining by anti-mouse IgG Alexa-546 (Red). Nuclei were stained blue with To-Pro-3 iodide. Images were examined using the 10× (A) and 63× (B) objective lens of a Zeiss LSM 780 meta confocal. (PDF) [file ppat.1004848.s007.pdf]

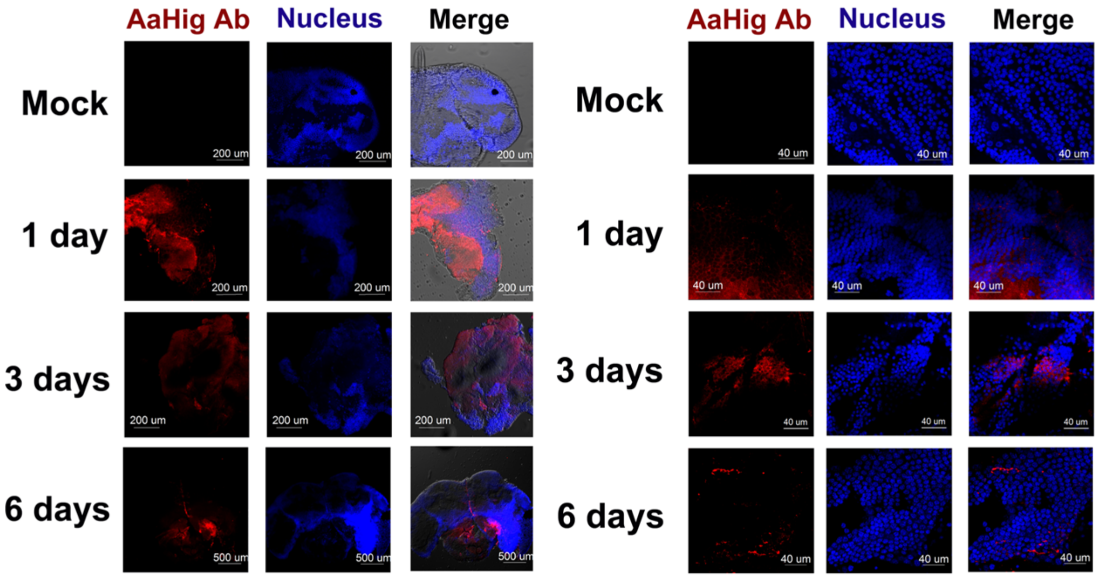

S7 Fig. The distribution of AaHig antibody in the *A. aegypti* brain

The 10-fold diluted murine AaHig antibody (Ab) was microinjected into the thorax of mosquitoes. At serial time points, the mosquito brains were fixed and dissected for staining by anti-mouse IgG Alexa-546 (Red). Nuclei were stained blue with To-Pro-3 iodide. Images were examined using the 10× (A) and 63× (B) objective lens of a Zeiss LSM 780 meta confocal.
