# Supplementary material for: A Neuron-Specific Antiviral Mechanism Prevents Lethal Flaviviral Infection of Mosquitoes
Source: PLoS Pathog. 2015 Apr 27;11(4):e1004848. doi: 10.1371/journal.ppat.1004848 (PMC4411065; doi:10.1371/journal.ppat.1004848)
Supplement: S9 Fig — The mosquitoes were fed with the Vero cells-generated DENV-2 and fresh human blood via Hemotek oral feeding system. Subsequently, the anti-AaHig antibody was intrathoracically microinjected into the mosquitoes 3 days after viral blood feeding. The fed mosquitoes that were inoculated by pre-immune antibody were used as a negative control. The mosquitoes were reared under standard condition. After removing the uninfected mosquitoes, the DENV load in mosquito bodies (A and B) and heads (C and D) was measured via qPCR at 6 days and 9 days after the oral infection. The primers and probes of qPCR were described in the S1 Table. The results were pooled from two parallel experiments. One dot represents 1 mosquito and the horizontal line represents the median value. The data were statistically analyzed by the non-parametric Mann-Whitney test. (PDF) [file ppat.1004848.s009.pdf]

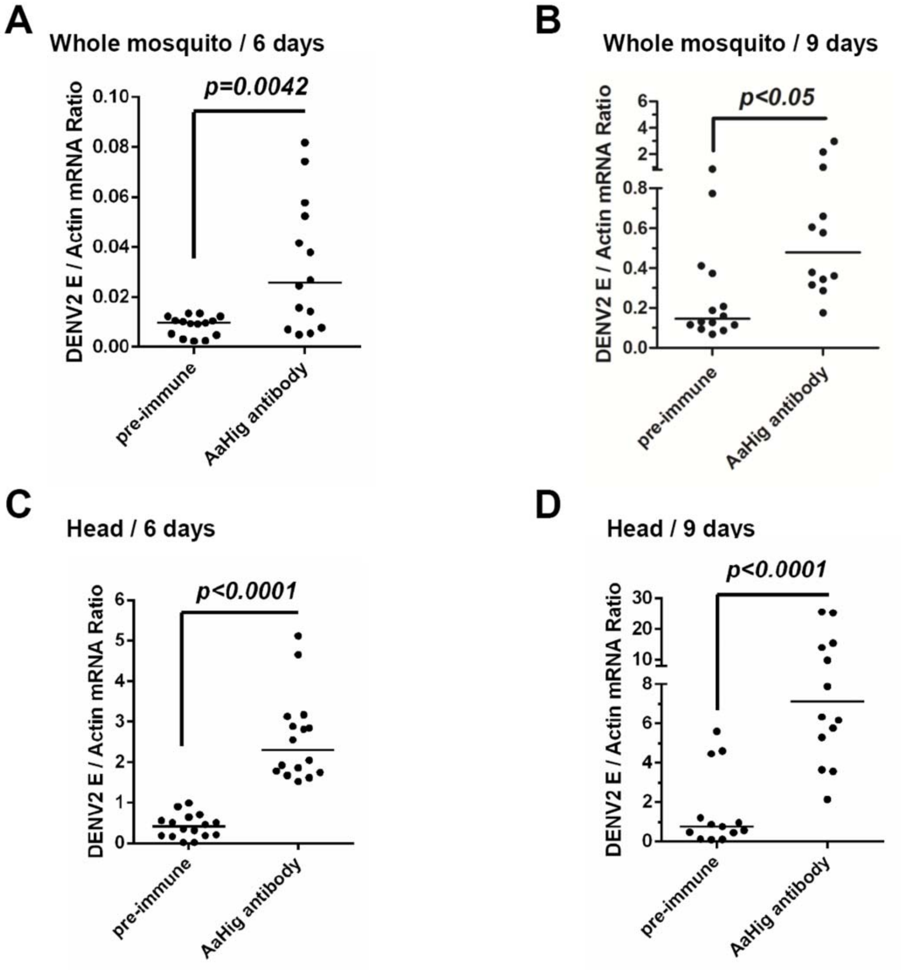

S9 Fig. Immuno-blockade of AaHig significantly enhanced the DENV replication in the mosquitoes infected by oral feeding

The mosquitoes were fed with the Vero cells-generated DENV-2 and fresh human blood via Hemotek oral feeding system. Subsequently, the anti-AaHig antibody was intrathoracically microinjected into the mosquitoes 3 days after viral blood feeding. The fed mosquitoes that were inoculated by pre-immune antibody were used as a negative control. The mosquitoes were reared under standard condition. After removing the uninfected mosquitoes, the DENV load in mosquito bodies (A and B) and heads (C and D) was measured via Taqman qPCR 6 days and 9 days after the oral infection. The primers and probes of qPCR were described in the S1 Table. The results were pooled from two parallel experiments. One dot represents 1 mosquito and the horizontal line represents the median value. The data were statistically analyzed by the non-parametric *Mann-Whitney* test.
