# Supplementary material for: A Neuron-Specific Antiviral Mechanism Prevents Lethal Flaviviral Infection of Mosquitoes
Source: PLoS Pathog. 2015 Apr 27;11(4):e1004848. doi: 10.1371/journal.ppat.1004848 (PMC4411065; doi:10.1371/journal.ppat.1004848)
Supplement: S10 Fig — The murine AaHig antibody with 10-fold dilutions was premixed with 10 M.I.D.50 DENV-2 to co-microinject into the mosquitoes. The salivary glands (A) and midguts (B) were then dissected to examine the viral load at 3 (i) and 6 (ii) days post-infection via TaqMan qPCR and normalized against A. aegypti actin. The results were combined from 2 independent experiments. The data were analyzed statistically using the non-parametric Mann-Whitney test. (PDF) [file ppat.1004848.s010.pdf]

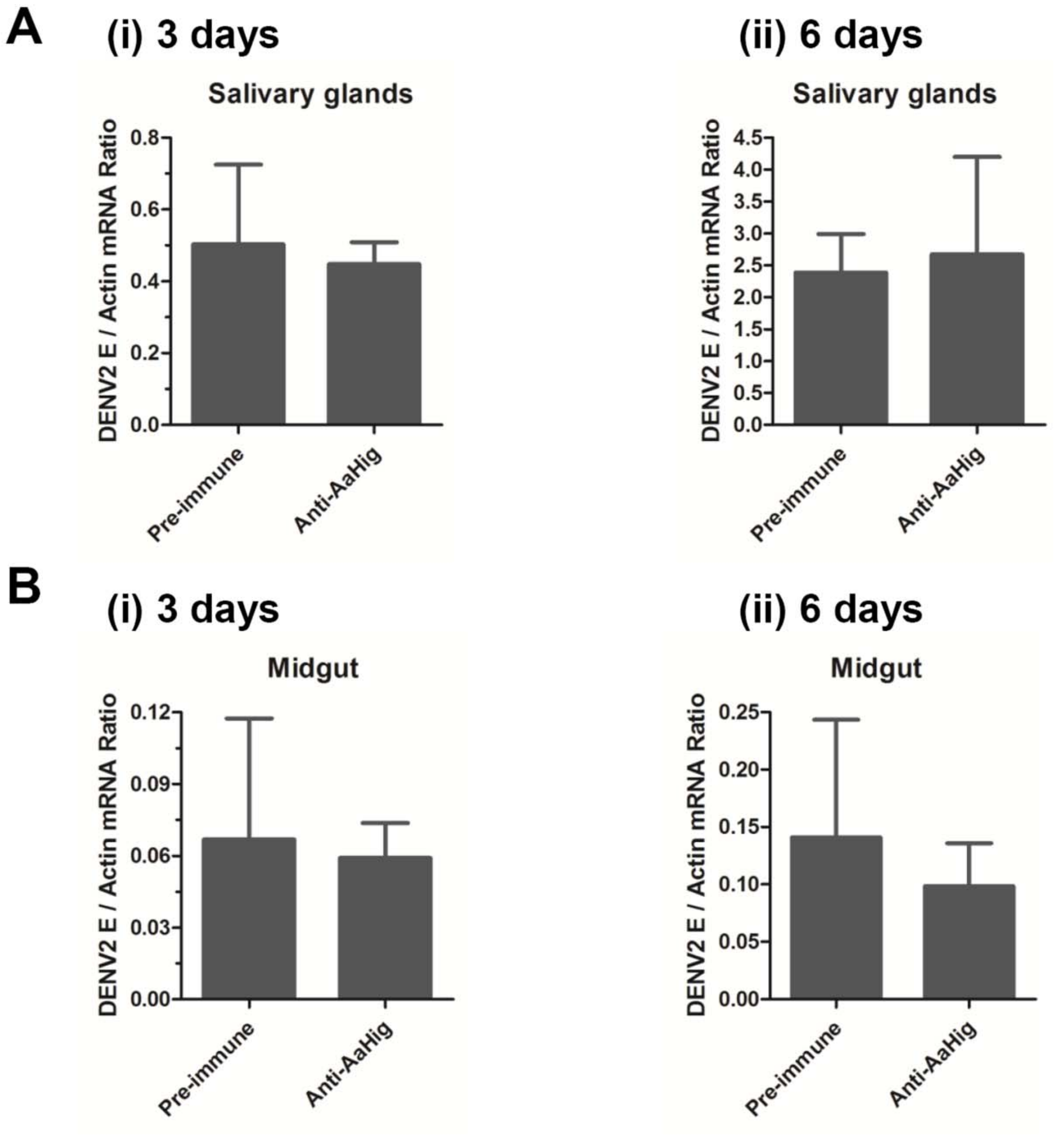

S10 Fig. Detection of viral burden in salivary glands and midguts of *A. aegypti*

The murine AaHig antibody with 10-fold dilutions was premixed with 10 M.I.D.$_{50}$ DENV-2 to co-microinject into the mosquitoes. The salivary glands (A) and midguts (B) were then dissected to examine the viral load at 3 (i) and 6 (ii) days post-infection via qPCR and normalized against *A. aegypti actin*. The results were combined from 2 independent experiments. The data were analyzed statistically using the non-parametric *Mann-Whitney* test.
