# Supplementary material for: A Neuron-Specific Antiviral Mechanism Prevents Lethal Flaviviral Infection of Mosquitoes
Source: PLoS Pathog. 2015 Apr 27;11(4):e1004848. doi: 10.1371/journal.ppat.1004848 (PMC4411065; doi:10.1371/journal.ppat.1004848)
Supplement: S11 Fig — (A-D) The effect of AaHig silencing in SINV infection of A. aegypti. 10 M.I.D.50 SINV were inoculated at 3 days post AaHig dsRNA inoculation. The viral load of whole bodies (A and B) and heads (C and D) was assessed 3 days and 6 days post-infection via qPCR and normalized with A. aegypti actin. The qPCR primers and probes were described in the S1 Table. The experiment was repeated two times with similar results. One dot represents 1 mosquito and the horizontal line represents the median value. The data were statistically analyzed by non-parametric Mann-Whitney test. (E) The expression of Sindbis Envelope proteins in Drosophila S2 cells. Three Sindbis Envelope genes (E1, E2 and E3) with FLAG tag were cloned into the pMT/Bip/V5-His A vector and expressed in the S2 cell supernatant. The supernatant from empty vector-transfected S2 cells was used as a mock. The E proteins were detected with an anti-FLAG antibody via western blotting. (F) The Sindbis E proteins do not interact with AaHig by a co-IP assay. Three Sindbis Envelope genes (E1, E2 and E3) were cloned into the pMT/Bip/V5-His A vector, and subsequently co-expressed with AaHig in the S2 cell supernatant. The protein complex was pulled down with an anti-FLAG antibody and detected using an anti-V5-HRP antibody. We reproduced the experiment 2 times. (PDF) [file ppat.1004848.s011.pdf]

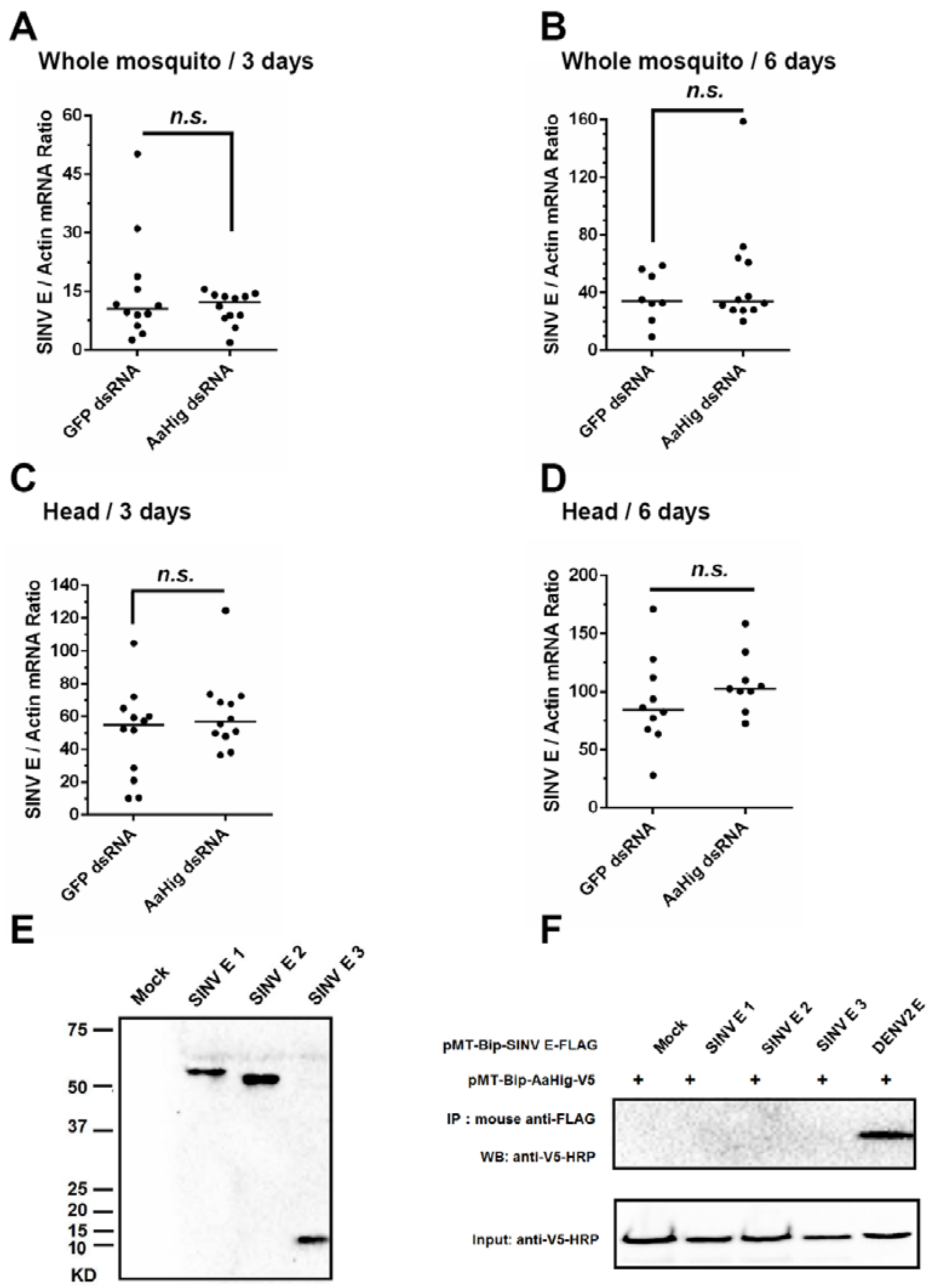

S11 Fig. *AaHig* silencing did not influence the SINV infection in *A. aegypti*

(A-D) The effect of *AaHig* silencing in SINV infection of *A. aegypti*. 10 M.I.D.$_{50}$ SINV were inoculated at 3 days post *AaHig* dsRNA inoculation. The viral load of whole bodies (A and B) and heads (C and D) was assessed at 3 days and 6 days post-infection via SYBR Green qPCR and normalized with *A. aegypti actin*. The qPCR primers and probes were described in the S1 Table. The experiment was repeated two times with similar results. One dot represents 1

mosquito and the horizontal line represents the median value. The data were statistically analyzed by the non-parametric *Mann-Whitney* test.

(E) The expression of Sindbis Envelope proteins in *Drosophila* S2 cells. Three Sindbis *Envelope* genes (E1, E2 and E3) with FLAG tag were cloned into the pMT/Bip/V5-His A vector and expressed in the S2 cell supernatant. The supernatant from empty vector-transfected S2 cells was used as a mock. The E proteins were detected with an anti-FLAG antibody via western blotting.

(F) The Sindbis E proteins do not interact with AaHig by a co-IP assay. Three Sindbis *Envelope* genes (E1, E2 and E3) were cloned into the pMT/Bip/V5-His A vector, and subsequently co-expressed with AaHig in the S2 cell supernatant. The protein complex was pulled down with an anti-FLAG antibody and detected using an anti-V5-HRP antibody. We reproduced the experiment 2 times.
