# Supplementary material for: A Neuron-Specific Antiviral Mechanism Prevents Lethal Flaviviral Infection of Mosquitoes
Source: PLoS Pathog. 2015 Apr 27;11(4):e1004848. doi: 10.1371/journal.ppat.1004848 (PMC4411065; doi:10.1371/journal.ppat.1004848)
Supplement: S12 Fig — The interaction of purified AaHig (V5) and JEV E (FLAG) proteins was determined by ELISA (A) and co-IP (B). In the ELISA detection, the binding was probed by mouse anti-FLAG-HRP antibody. The data were presented as the mean ± standard error. The experiment was reproduced 3 times. In the co-IP assay, the protein complex was pulled down with an anti-V5 antibody and detected using a mouse anti-FLAG-HRP antibody. We reproduced the experiments 3 times. (PDF) [file ppat.1004848.s012.pdf]

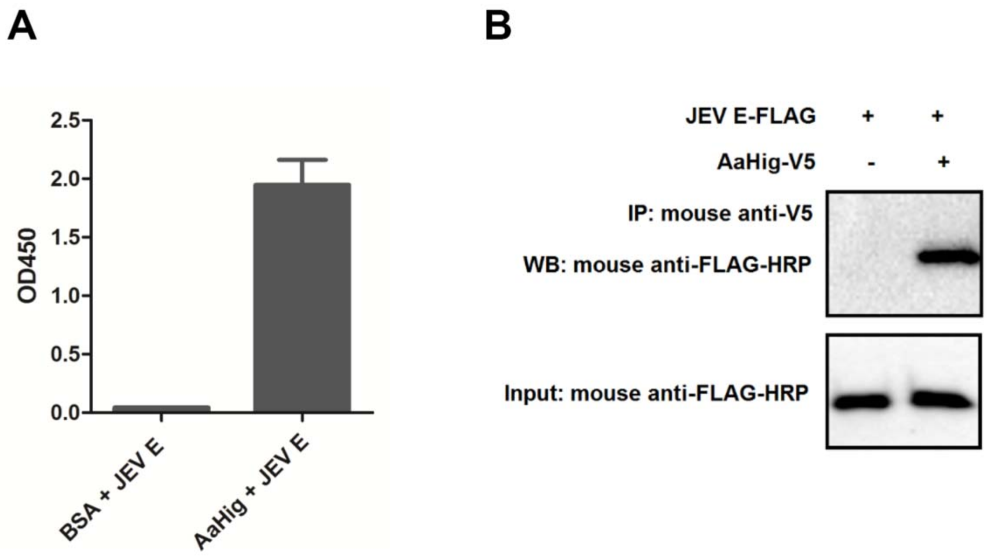

S12 Fig. The interaction between AaHig and JEV E protein

The interaction of purified AaHig (V5) and JEV E (FLAG) proteins was determined by ELISA (A) and co-IP (B). In the ELISA detection, the binding was probed by mouse anti-FLAG-HRP antibody. The data were presented as the mean ± standard error. The experiment was reproduced 3 times. In the co-IP assay, the protein complex was pulled down with an anti-V5 antibody and detected using a mouse anti-FLAG-HRP antibody. We reproduced the experiment 3 times.
