# Supplementary material for: A Neuron-Specific Antiviral Mechanism Prevents Lethal Flaviviral Infection of Mosquitoes
Source: PLoS Pathog. 2015 Apr 27;11(4):e1004848. doi: 10.1371/journal.ppat.1004848 (PMC4411065; doi:10.1371/journal.ppat.1004848)
Supplement: S13 Fig — The seven truncations, in which the functional domains of AaHig were sequentially deleted, were expressed in mosquito Aag2 cells. The membrane location of these truncations was determined by immunofluorescence staining. The AaHig truncations were stained with an anti-V5 antibody (Red); the cellular membrane was stained by a plasma membrane marker, Wheat Germ Agglutinin (WGA) (Green); nuclei were stained blue with To-Pro-3 iodide (Blue). Images were examined using a Zeiss LSM 780 meta confocal 63×objective lens. (PDF) [file ppat.1004848.s013.pdf]

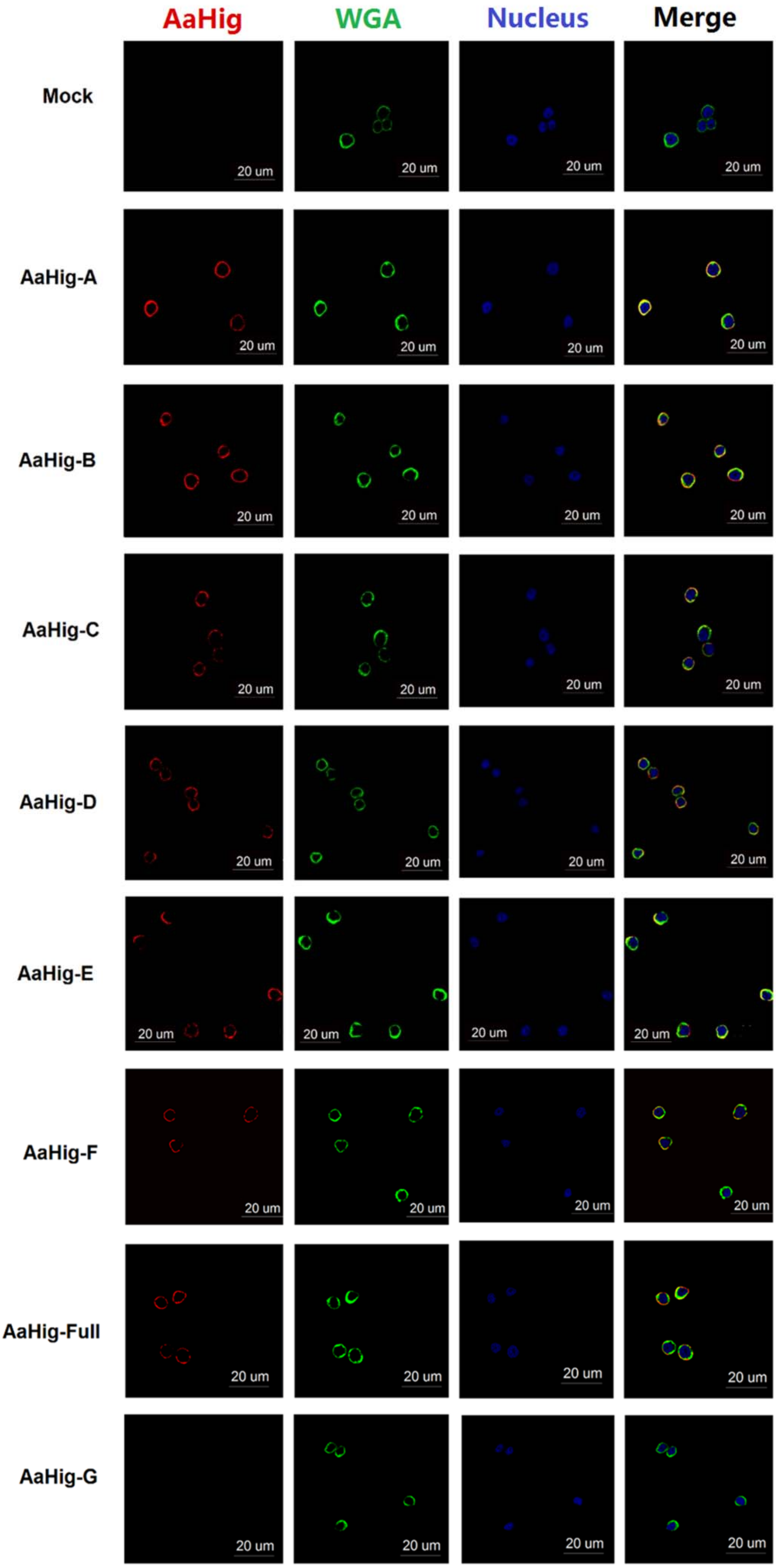
AaHig
WGA
Nucleus
Merge
Mock
AaHig-A
AaHig-B
AaHig-C
AaHig-D
AaHig-E
AaHig-F
AaHig-Full
AaHig-G
20 um

S13 Fig. The membrane-bound capability of AaHig truncations on mosquito Aag2 cells

The seven truncations, in which the functional domains of AaHig were sequentially deleted, were expressed in mosquito Aag2 cells. The membrane location of these truncations was determined by immunofluorescence staining. The AaHig truncations were stained with an anti-V5 antibody (Red); the cellular membrane was stained by a plasma membrane marker, Wheat Germ Agglutinin (WGA) conjugated with Alexa Fluor-488 (Green); nuclei were stained blue with To-Pro-3 iodide (Blue). Images were examined using a Zeiss LSM 780 meta confocal 63×objective lens.
