# Supplementary material for: A Neuron-Specific Antiviral Mechanism Prevents Lethal Flaviviral Infection of Mosquitoes
Source: PLoS Pathog. 2015 Apr 27;11(4):e1004848. doi: 10.1371/journal.ppat.1004848 (PMC4411065; doi:10.1371/journal.ppat.1004848)
Supplement: S14 Fig — (A-B) AaHig did not reduce the DENV infection in the mammalian cells. 2μg AaHig purified protein and 0.01 M.O.I. DENV-2 was incubated with human A549 (A) and Vero (B) cells. The same amount of BSA with DENV-2 was used as negative controls. After 48 hours, the viral load was determined by Taqman qPCR and normalized by human GAPDH. The data were presented as the mean ± standard error. The results were combined from 2 independent experiments. (C) The ectopic expression of Drosophila melanogaster Hig (DmHig) in S2 cells. The gene of DmHig were cloned into the pAc5.1/V5-His A vector and expressed in the S2 cell supernatant. The supernatant from empty vector-transfected S2 cells was used as a mock. The recombinant protein was detected with an anti-V5 antibody via western blotting. (D-E) The antiviral effect of DmHig in insect cells. DmHig was overexpressed in mosquito Aag2 (D) and Drosophila S2 (E) cells. After 48 hrs, the 0.01 M.O.I. DENV-2 was added into the cells, and subsequently the viral load was determined by Taqman qPCR and normalized by A. aegypti and D. melanogaster actins. The data were presented as the mean ± standard error. The results were combined from 3 independent experiments. (PDF) [file ppat.1004848.s014.pdf]

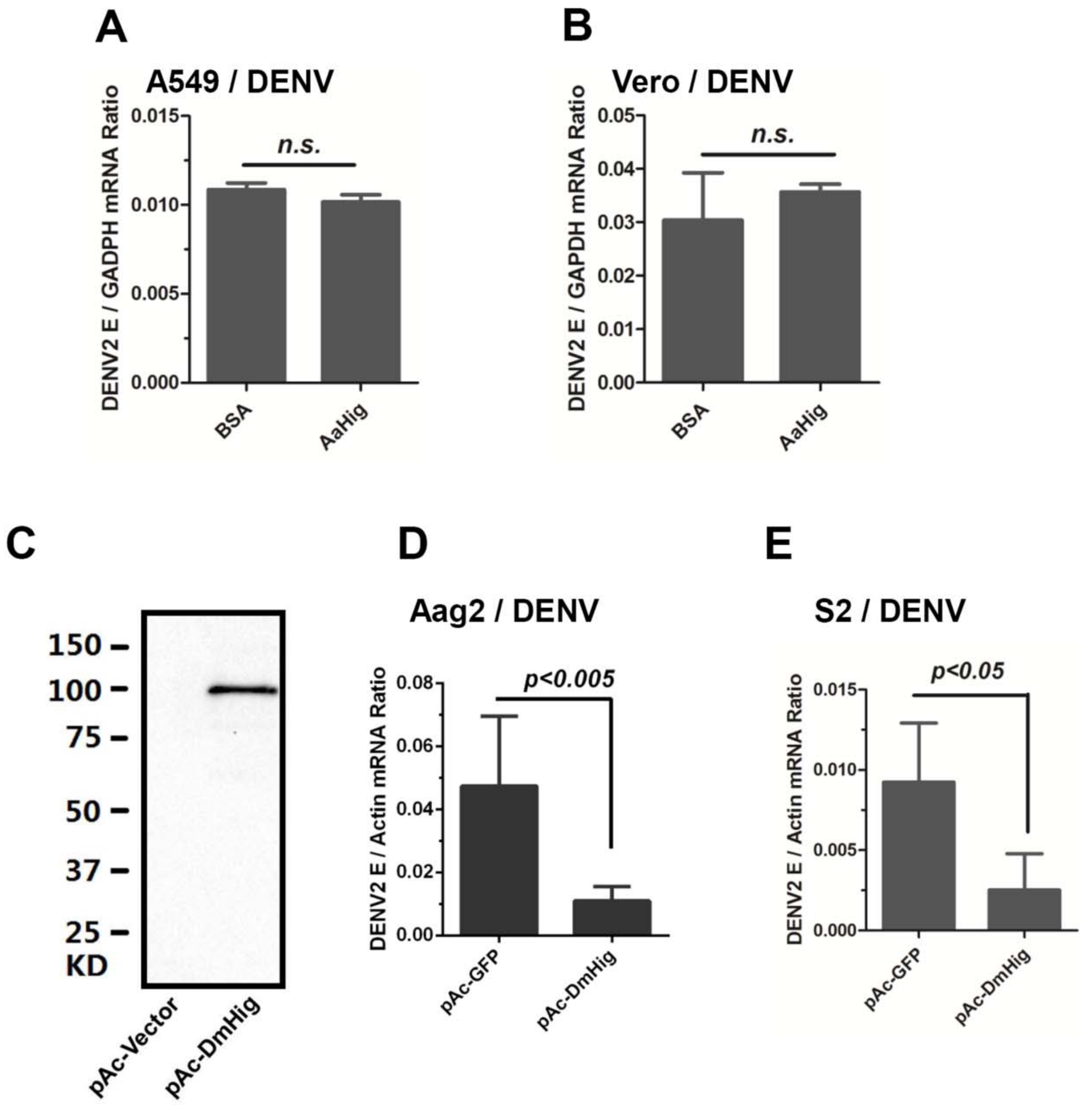

S14 Fig. The antiviral effect of Hig protein in mammalian cells and insect cells

(A-B) AaHig did not reduce the DENV infection in the mammalian cells. 2μg AaHig purified protein and 0.01 M.O.I. DENV-2 was incubated with human A549 (A) and Vero (B) cells. The same amount of BSA with DENV-2 was used as a negative control. After 48 hrs, the viral load was determined by Taqman qPCR and normalized by human *GAPDH*. The data were presented as the mean ± standard error. The results were combined from 2 independent experiments.

(C) The ectopic expression of *Drosophila melanogaster Hig* (*DmHig*) in S2 cells. The gene of *DmHi*g were cloned into the pAc5.1/V5-His A vector and expressed in the S2 cell supernatant.

The supernatant from empty vector-transfected S2 cells was used as a mock. The recombinant protein was detected with an anti-V5 antibody via western blotting.

(D-E) The antiviral effect of *DmHig* in insect cells. DmHig was overexpressed in mosquito Aag2 (D) and *Drosophila* S2 (E) cells. After 48 hrs, the 0.01 M.O.I. DENV-2 was added into the cells, and subsequently the viral load was determined by Taqman qPCR and normalized by *A. aegypti* and *D. melanogaster actins*. The data were presented as the mean ± standard error. The result were combined from 3 independent experiments.
