# Supplementary material for: A Neuron-Specific Antiviral Mechanism Prevents Lethal Flaviviral Infection of Mosquitoes
Source: PLoS Pathog. 2015 Apr 27;11(4):e1004848. doi: 10.1371/journal.ppat.1004848 (PMC4411065; doi:10.1371/journal.ppat.1004848)
Supplement: S15 Fig — The 5 ug purified AaHig protein was incubated with the Vero cells for 1 hr. After 5 washing by PBS buffer, the cells were fixed by 4% PFA and staining by AaHig antibody. The WGA conjugated with Alexa Fluor-488 was used to stain the cellular membrane. The Aag2 cells with the same amount of AaHig served as a positive control. (PDF) [file ppat.1004848.s015.pdf]

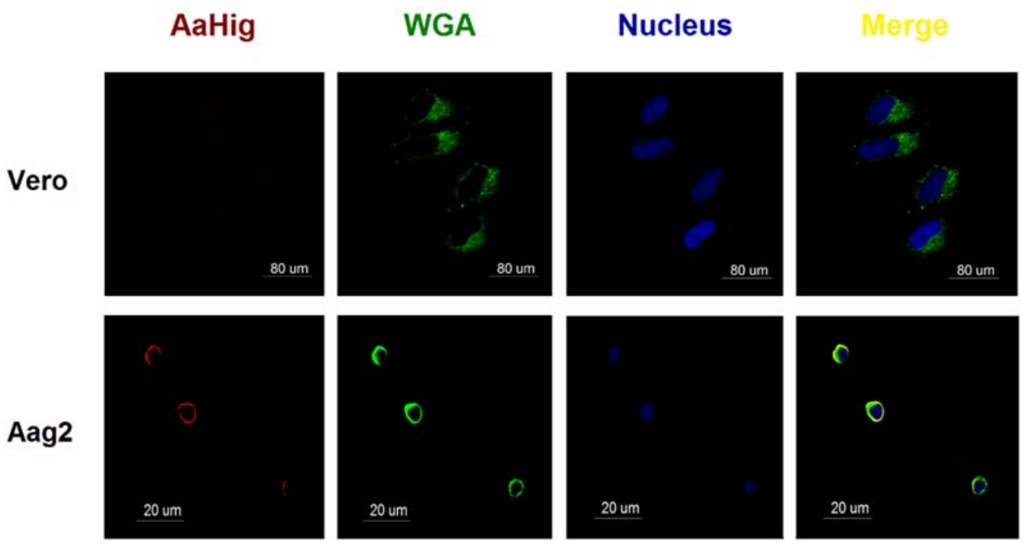

S15 Fig. AaHig cannot coat on the membrane of Vero cells by an immuno-staining assay

The 5μg purified AaHig protein was incubated with the Vero cells for 1 hr. After 5 washing by PBS buffer, the cells were fixed by 4% PFA and staining by AaHig antibody. The WGA conjugated with Alexa Fluor-488 was used to stain the cellular membrane. The Aag2 cells with the same amount of AaHig served as a positive control.
