# Supplementary material for: A Neuron-Specific Antiviral Mechanism Prevents Lethal Flaviviral Infection of Mosquitoes
Source: PLoS Pathog. 2015 Apr 27;11(4):e1004848. doi: 10.1371/journal.ppat.1004848 (PMC4411065; doi:10.1371/journal.ppat.1004848)
Supplement: S16 Fig — The murine AaHig antibody with 10-fold dilution was microinjected in A. aegypti. The same amount of diluted pre-immune antibody was used as a negative control. The expression of immune-related genes, such as AMPs (A-C), A. aegypti Ago2 (AaAgo2) (D), A. aegypti Dicer2 (AaDicer 2) (E), A. aegypti Duox1 (AaDuox1) (F) and A. aegypti Duox2 (AaDuox2) (G), was determined by qPCR at 6 hrs post-inoculation in mosquitoes. The amount of genes was normalized by A. aegypti actin. (PDF) [file ppat.1004848.s016.pdf]

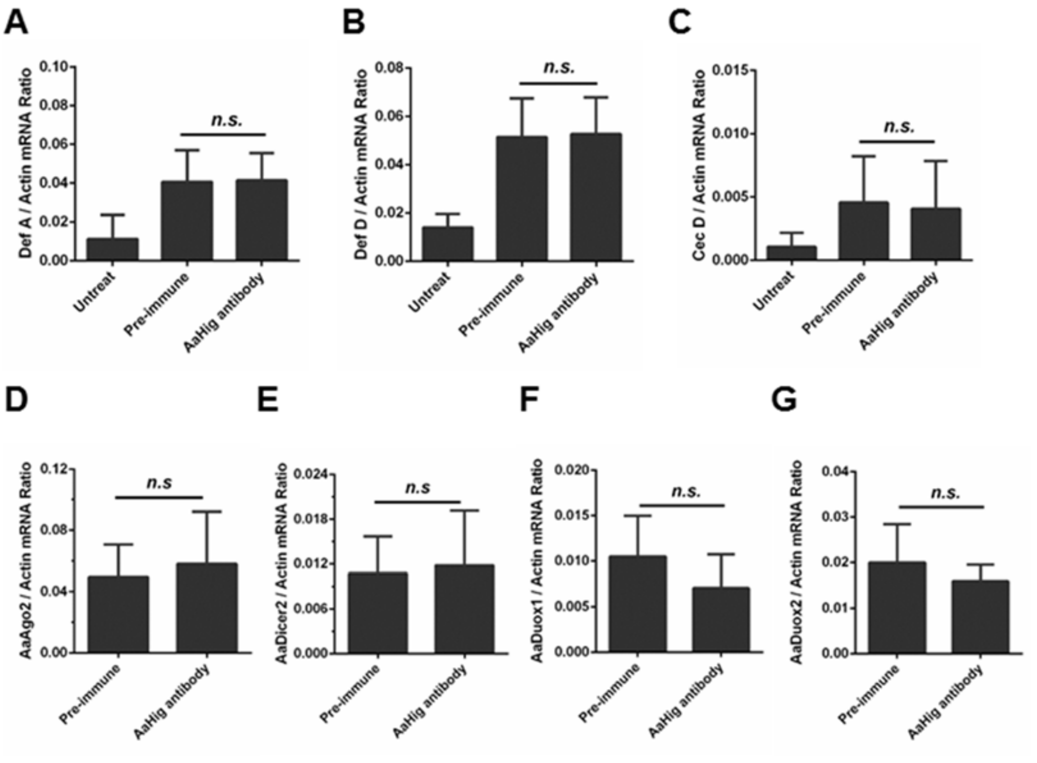

S16 Fig. Immuno-blockade of AaHig does not alter the expression of immune-related genes

The murine AaHig antibody with 10-fold dilution was microinjected in *A. aegypti*. The same amount of diluted pre-immune antibody was used as a negative control. The expression of immune-related genes, such as *AMPs* (A-C), *A. aegypti Ago2* (*AaAgo2*) (D), *A. aegypti Dicer2* (*AaDicer 2*) (E), *A. aegypti Duox1* (*AaDuox1*) (F) and *A. aegypti Duox2* (*AaDuox2*) (G), was determined by qPCR at 6 hrs post-inoculation in mosquitoes. The amount of genes was normalized by *A. aegypti actin.*
