# Supplementary material for: A Neuron-Specific Antiviral Mechanism Prevents Lethal Flaviviral Infection of Mosquitoes
Source: PLoS Pathog. 2015 Apr 27;11(4):e1004848. doi: 10.1371/journal.ppat.1004848 (PMC4411065; doi:10.1371/journal.ppat.1004848)
Supplement: S17 Fig — (A-B) Overexpression of AaHig did not regulate the melanization activity in Aag2 cells. The phenoloxidase (PO) activity was measured in DENV-2 (A) or JEV (B) infected or mock cells. The regulation of PO activity was presented by the fold change than that in uninfected pAc-GFP-transfected mock cells. (C) Knockdown of AaHig did not influence the H2O2 release in various mosquito parts. The dsRNA mediated silencing was performed in mosquitoes. The same amount of GFP dsRNA was inoculated into mosquito thorax as a negative control. At 3 days later, 1000 M.I.D.50 DENV-2 were microinjected into mosquitoes. The various mosquito parts were collected at 6 hrs post infection. The concentration of H2O2 was measured by a Hydrogen Peroxide Assay Kit. The result was presented by the fold change calculated by H2O2 concentration in the infected mosquitoes / that in uninfected mosquitoes. (PDF) [file ppat.1004848.s017.pdf]

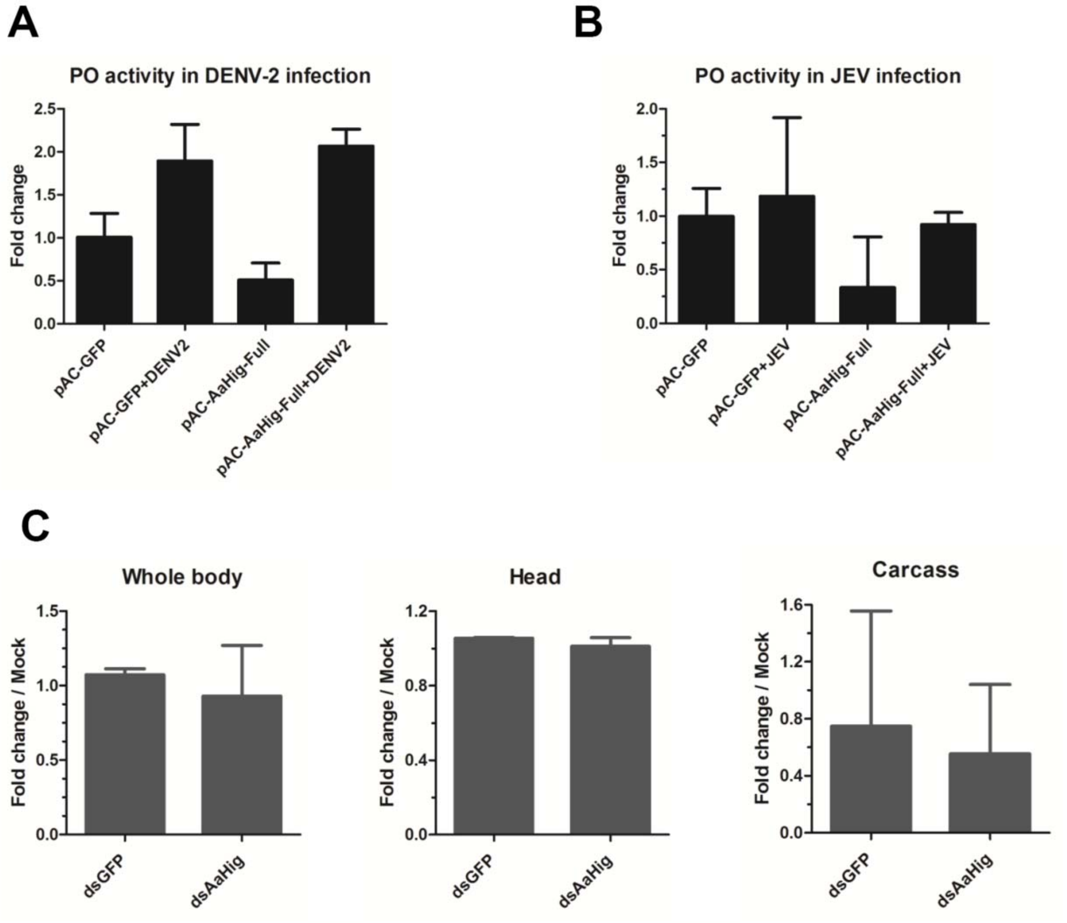

S17 Fig. The role of AaHig in the activation of melanization and reactive oxygen systems

(A-B) Overexpression of AaHig did not regulate the melanization activity in Aag2 cells. The phenoloxidase (PO) activity was measured in DENV-2 (A) or JEV (B) infected or mock cells. The regulation of PO activity was presented by the fold change than that in uninfected pAc-GFP-transfected mock cells.

(C) Knockdown of *AaHig* did not influence the H_2O_2 release in various mosquito parts. The dsRNA mediated silencing was performed in mosquitoes. The same amount of *GFP* dsRNA was inoculated into mosquito thorax as a negative control. At 3 days later, 1000 M.I.D.$_{50}$ DENV-2 were microinjected into mosquitoes. The various mosquito parts were collected at 6 hrs post infection. The concentration of H_2O_2 was measured by a Hydrogen Peroxide Assay Kit. The result was presented by the fold change calculated by H_2O_2 concentration in the infected mosquitoes / that in the uninfected mosquitoes.
