# Supplementary material for: A Neuron-Specific Antiviral Mechanism Prevents Lethal Flaviviral Infection of Mosquitoes
Source: PLoS Pathog. 2015 Apr 27;11(4):e1004848. doi: 10.1371/journal.ppat.1004848 (PMC4411065; doi:10.1371/journal.ppat.1004848)
Supplement: S18 Fig — (A-B) JEV attachment assay at 4°C. The serial concentration of purified AaHig protein was premixed with 5 M.O.I. JEV on ice, and then the Aag2 (A) and C6/36 (B) cells were incubated with the mixture for a time course at 4°C. The cells were washed 5 times by cold PBS buffer and collected at certain time points for total RNA isolation. (C-D) JEV internalization assay. The serial concentration of purified AaHig protein was premixed with 5 M.O.I. JEV, and consequently the mixture was incubated into the Aag2 cells at 28°C (C) and C6/36 cells at 30°C (D). The cells were washed 5 times by PBS at room temperature and collected for detection. (A-D) For the assay at 48 hrs, the cells were washed 5 times after 1 hr incubation at 4°C (A and B) or 28°C/30°C (C and D), and consequently cultured at 28°C or 30°C for an additional 48 hrs. The viral genome was determined by Taqman qPCR and normalized by A. aegypti actin. The data were presented as the mean ± standard error. The results were combined from 3 independent experiments. *, p<0.05; **, p<0.01; ***, p<0.001. (PDF) [file ppat.1004848.s018.pdf]

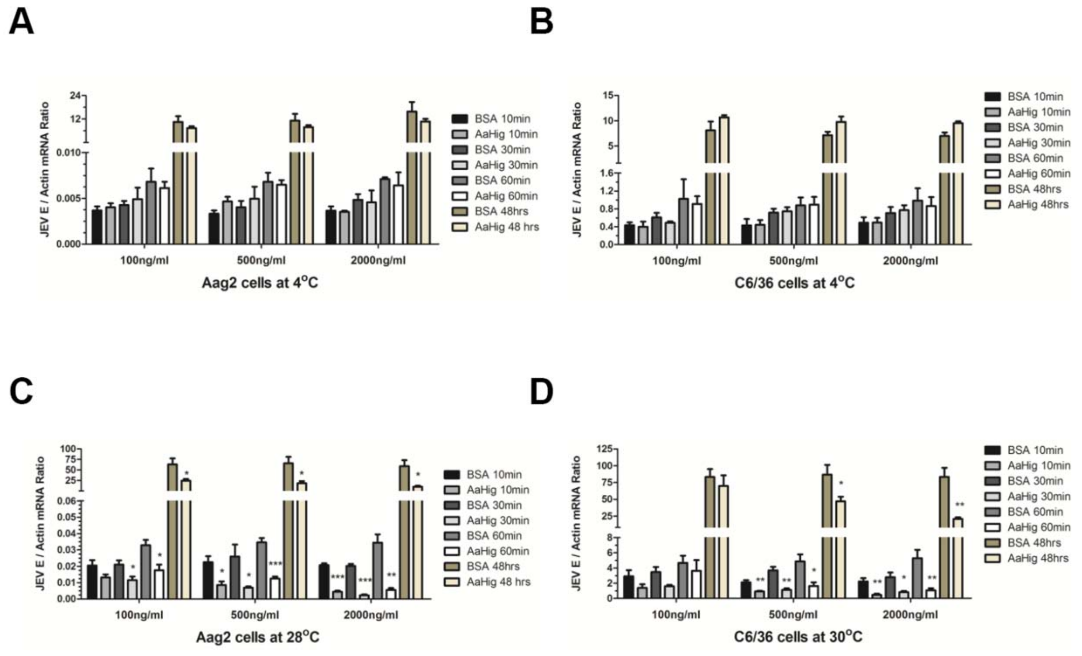

S18 Fig. AaHig reduces JEV entry in mosquito cells

(A-B) JEV attachment assay at 4 °C. The serial concentration of purified AaHig protein was premixed with 5 M.O.I. JEV on ice, and then the Aag2 (A) and C6/36 (B) cells were incubated with the mixture for a time course at 4°C. The cells were washed 5 times by cold PBS buffer and collected at certain time points for total RNA isolation.

(C-D) JEV internalization assay. The serial concentration of purified AaHig protein was premixed with 5 M.O.I. JEV, and consequently the mixture was incubated into the Aag2 cells at 28°C (C) and C6/36 cells at 30°C (D). The cells were washed 5 times by PBS at room temperature and collected for detection.

(A-D) For the assay at 48 hrs, the cells were washed 5 times after 1 hr incubation at 4°C (A and B) or 28°C/30°C (C and D), and consequently cultured at 28°C or 30°C for an additional 48 hrs. The viral genome was determined by Taqman qPCR and normalized by *A. aegypti actin*. The data were presented as the mean ± standard error. The results were combined from 3 independent experiments. *, *p<0.05*; **, *p<0.01*; ***, *p<0.001*.
