# Supplementary material for: A Neuron-Specific Antiviral Mechanism Prevents Lethal Flaviviral Infection of Mosquitoes
Source: PLoS Pathog. 2015 Apr 27;11(4):e1004848. doi: 10.1371/journal.ppat.1004848 (PMC4411065; doi:10.1371/journal.ppat.1004848)
Supplement: S19 Fig — The nano-beads with a 10–100 nm diameter, which mimic the size of viruses, were selected to perform a particle uptake assay in mosquito Aag2 cells. The beads were pre-labeled by Fluorescein isothiocyanat (FITC). AaHig protein was premixed with the beads, then incubated the mixture with Aag2 cells at 28°C for 30 min. The same amount of BSA mixed with the beads was used as a negative control. The mock group was the cells without the beads. After the incubation, the cells were washed 3 times by PBS buffer, and then treated by 0.2%Trypan Blue to quench the fluorescence of the beads attached on the cell surface. The amount of uptake beads, which had been internalized into mosquito cells, was measured by the flow cytometry. We reproduced these experiments 3 times. (PDF) [file ppat.1004848.s019.pdf]

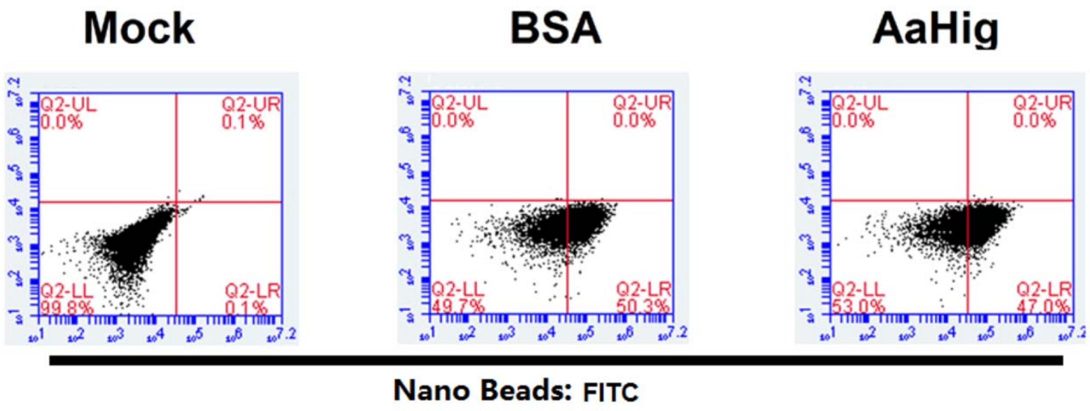

S19 Fig. AaHig does not generally sequester unrelated particles on plasma membrane

The nano-beads with a 10-100 nm diameter, which mimic the size of the viruses, were selected to perform a particle uptake assay in mosquito Aag2 cells. The beads were pre-labeled by Fluorescein isothiocyanat (FITC). AaHig protein was premixed with the beads, then incubated the mixture with Aag2 cells at 28°C for 30 min. The same amount of BSA mixed with the beads was used as a negative control. The mock group was the cells without the beads. After the incubation, the cells were washed 3 times by PBS buffer, and then treated by 0.2%Trypan Blue to quench the fluorescence of the beads attached on the cell surface. The amount of uptake beads, which had been internalized into mosquito cells, was measured by the flow cytometry. We reproduced these experiments 3 times.
