# Supplementary material for: A Neuron-Specific Antiviral Mechanism Prevents Lethal Flaviviral Infection of Mosquitoes
Source: PLoS Pathog. 2015 Apr 27;11(4):e1004848. doi: 10.1371/journal.ppat.1004848 (PMC4411065; doi:10.1371/journal.ppat.1004848)
Supplement: S20 Fig — The 2 μg AaHig protein was premixed with 5 M.O.I. Sindbis virus (SINV), and then we incubated the materials with human A549 cells (A) at 37°C or mosquito Aag2 cells (B) at 28°C for a serial time course. The same amount of BSA mixed with viruses was used as a mock control. The SINV load was determined by qPCR and normalized by human or mosquito actins. The experiment was repeated by three times with the similar results. (PDF) [file ppat.1004848.s020.pdf]

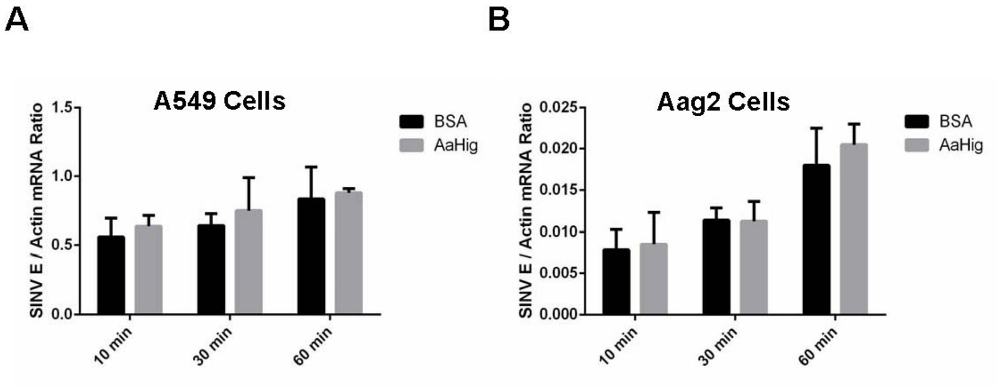

S20 Fig. Over-expression of *AaHig* does not influence the Sindbis burden in the human and mosquito cells

The 2μg AaHig protein was premixed with 5 M.O.I. Sindbis virus (SINV), and then we incubated the materials with human A549 cells (A) at 37 ^{o}C or mosquito Aag2 cells (B) at 28 ^{o}C for a serial time course. The same amount of BSA mixed with viruses was used as a mock control. The SINV load was determined by qPCR and normalized by human or mosquito *actins*. The experiment was repeated by three times with the similar results.
