# Supplementary material for: A Neuron-Specific Antiviral Mechanism Prevents Lethal Flaviviral Infection of Mosquitoes
Source: PLoS Pathog. 2015 Apr 27;11(4):e1004848. doi: 10.1371/journal.ppat.1004848 (PMC4411065; doi:10.1371/journal.ppat.1004848)
Supplement: S21 Fig — A rabbit anti-Drosophila Rab5 polyclonal antibody can efficiently probe Rab5 proteins in the lysates of S2 and Aag2 cells. (PDF) [file ppat.1004848.s021.pdf]

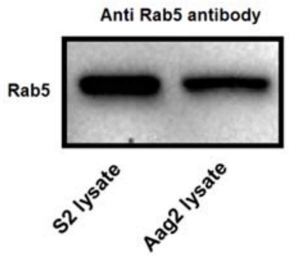

S21 Fig. Validation of *Drosophila* Rab5 polyclonal antibody

A rabbit anti-*Drosophila* Rab5 polyclonal antibody can efficiently probe Rab5 proteins in the lysates of S2 and Aag2 cells.
