# Supplementary material for: A Neuron-Specific Antiviral Mechanism Prevents Lethal Flaviviral Infection of Mosquitoes
Source: PLoS Pathog. 2015 Apr 27;11(4):e1004848. doi: 10.1371/journal.ppat.1004848 (PMC4411065; doi:10.1371/journal.ppat.1004848)
Supplement: S1 Table — (PDF) [file ppat.1004848.s022.pdf]

S1 Table Primers and probes for qPCR, dsRNA synthesis and genes cloning

**Primers for cloning into pAc5.1/V5-His/A**	**Upper primer**	**Lower primer**	
AaHig-A (V5 tag in the C-terminal)	AGAGCGGCCGCGCCACCATGCCTCGAACGACGGTG	AGACTCGAGCTCTGGGCTTTCTTCGATCTC	
AaHig-B (V5 tag in the C-terminal)	AGAGCGGCCGCGCCACCATGCCTCGAACGACGGTG	AGACTCGAGTGATGGTGGACTGTCGTCAGAG	
AaHig-C (V5 tag in the C-terminal)	AGAGCGGCCGCGCCACCATGCCTCGAACGACGGTG	AGACTCGAG CGATATGGCCACAACGATC	
AaHig-D (V5 tag in the C-terminal)	AGAGCGGCCGCGCCACCATGCCTCGAACGACGGTG	AGACTCGAG GCAGTTGGGAGTCGGCGATG	
AaHig-E (V5 tag in the C-terminal)	AGAGCGGCCGCGCCACCATGCCTCGAACGACGGTG	AGACTCGAG ACATCTGGGAATAGGGCCAC	
AaHig-F (V5 tag in the C-terminal)	AGAGCGGCCGCGCCACCATGCCTCGAACGACGGTG	AGACTCGAG ACAGAACGGCGGAGGATGGG	
AaHig-Full (V5 tag in the C-terminal)	AGAGCGGCCGCGCCACCATGCCTCGAACGACGGTG	GGACTCGAGAATCTCTTCATAACTTCGACA	
**Primers for cloning into pMT/BiP/V5-His/A**	**Upper primer**	**Lower primer**	
DENV-2 Envelop gene (3×FLAG tag in N-terminal)	CGAAGATCTGCCACCATGGACTACAAAGACCATGACGGTGATTATAAAGATCATGACATCGATTACAAGGATGACGATGACAAGATGCGTTGCATAGGAATA	AGACTCGAGTCAATGGTGATGGTGATGATGTTGGCCGATAGAACTTC	
DENV-2 Envelop gene (1-52aa)(3×FLAG tag in N-terminal)	GGAAGATCTGACTACAAAGACCATGACGGTGATTATAAAGATCATGACATCGATTACAAGGATGACGATGACAAGATGCGTTGCATAGGAATATCA	CCGCTCGAGTCATTGTTTGGCTTCTGTTTC	
DENV-2 Envelop gene (1-132aa)(3×FLAG tag in N-terminal)	GGAAGATCTGACTACAAAGACCATGACGGTGATTATAAAGATCATGACATCGATTACAAGGATGACGATGACAAGATGCGTTGCATAGGAATATCA	CCGCTCGAGTCATGGTTGCACGACTTTTCC	
DENV-2 Envelop gene (1-193aa)(3×FLAG tag in N-terminal)	GGAAGATCTGACTACAAAGACCATGACGGTGATTATAAAGATCATGACATCGATTACAAGGATGACGATGACAAGATGCGTTGCATAGGAATATCA	CCGCTCGAGTCAGAAGTCGAGGCCCGTTCT	
DENV-2 Envelop gene (1-280aa)(3×FLAG tag in N-terminal)	GGAAGATCTGACTACAAAGACCATGACGGTGATTATAAAGATCATGACATCGATTACAAGGATGACGATGACAAGATGCGTTGCATAGGAATATCA	CCGCTCGAGTCATGTGAACAGTAAGTTTCC	
DENV-2 Envelop gene (1-296aa)(3×FLAG tag in N-terminal)	GGAAGATCTGACTACAAAGACCATGACGGTGATTATAAAGATCATGACATCGATTACAAGGATGACGATGACAAGATGCGTTGCATAGGAATATCA	CCGCTCGAGTCATCCTTTGAGCTGTAGTTT	
DENV-2 Envelop gene (297-400aa)(3×FLAG tag in N-terminal)	GGAAGATCTGATTACAAGGATGACGATGACAAGATGTCATACTCTATGTGC	CCGCTCGAGTCATTGGCCGATAGAACTTCC	
JEV Envelop gene (3×FLAG tag in N-terminal)	CGAAGATCTGCCACCATGGACTACAAAGACCATGACGGTGATTATAAAGATCATGACATCGATTACAAGGATGACGATGACAAGTTTAATTGTCTGGGAATGGGC	AGACTCGAGTCAATGGTGATGGTGATGATGAAGGCACCACCAAACACT	
AaHig-Full (V5 tag in the C-terminal)	CAACAGATCTGCCACCATGCCTCGAACGACGGTG	GGACTCGAGAATCTCTTCATAACTTCGACA	
AaHig-G (V5 tag in the C-terminal)	CAACAGATCTCTTCATCAATTCCGGGAGG	GGACTCGAGAATCTCTTCATAACTTCGACA	
SINV E1 (3×FLAG tag in the C-terminal)	CCCTCAAGATCTTACGAACATGCGACCACT	CCCTCATCTAGACTTGTCATCGTCATCCTTGTAATCGATGTCATGATCTTTATAATCACCGTCATGGTCTTTGTAGTCTGAAGTTTTTGAGATGGCGG	
SINV E2 (3×FLAG tag in the C-terminal)	CCCTCAAGATCTAAAAGAAGCGTCACTGACGA	CCCTCATCTAGACTTGTCATCGTCATCCTTGTAATCGATGTCATGATCTTTATAATCACCGTCATGGTCTTTGTAGTCGGTGTACACAGGATGGCG	
SINV E3 (3×FLAG tag in the C-terminal)	CCCTCAAGATCTTCTGCTGCACCACTGGTC	CCCTCATCTAGACTTGTCATCGTCATCCTTGTAATCGATGTCATGATCTTTATAATCACCGTCATGGTCTTTGTAGTCTTTTTTACTTCTGCCGGA	
**The primers for genes cloning(pET28)**	**Upper primer**	**Lower primer**	
AaHig-Full	TATCTAGCTAGCCGATCAGCGTTCGATAC	TCACCGCTCGAGAATCTCTTCATAACTTC	
**The primers for RT-qPCR**	**Upper primer**	**Lower primer**	**Probe (for Taqman QPCR)**
AaHig	GAGTAGAGCAAAGGGAAGTGG	CTCCTCATCTGGACCTTCGTG	
DENV-2 Envelop gene	CATTCCAAGTGAGAATCTCTTTGTCA	CAGATCTCTGATGAATAACCAACG	FAM-ATGCTGAAACGCGAGAGAAACCGC-TAMRA
JEV Envelop gene	CTGGTCCATAGGGAGTGGTTTC	CTCCACGCTGTGCTCGAA	FAM-TGACCTCGCTCTCCCCTGGACG-TAMRA
SINV Envelop gene	CGGCTATGGCAGGTTCGTA	CGCGCTTCAAGGACTTTTTC	
Aedes Aegypti Actin	GAACACCCAGTCCTGCTGACA	TGCGTCATCTTCTCACGGTTAG	FAM-AGGCCCCGCTCAACCCGAAG-TRAMA
CpHig	CCGAGGTGCGTTTCCAGG	GTCCTGTTCGTGCTCGTTCATC	
Culex Actin	TACGAACTTCCCGACGGA	GATACCGCACGATTCCATA	
Human GAPDH	AGCCTCAAGATCATCAGCAATG	ATGGACTGTGGTCATGAGTCCTT	
Drosophila melanogaster Actin	CCCAAGGCCAACCGTGAGAA	CGGAGGCGTACAGCGAGAGC	
AaDef A	CTATCAGGCTGCCGTGGAG	CAATGAGCAGCACAAGCACTATC	
AaDef D	GGCGTTGGTGATAGTGCTTG	CACACCTTCTTGGAGTTGCAG	
AaCec D	GAAGAAGCTGGGAAAGAAATTG	CCAATCGCTTTTATTCCTACAAC	
AaAgo2	AGGTAATCAACGCCAAAACGAACG	AAGCGGCCACTCCCACCACACT	
AaDicer	AATCATTCCGCCCGAGTGCTAT	GTCCCCCATGGTCTGCTGTGA	
AaDuox1	ATGCTGAGCCCAGAGAGATT	TTTCCTCATCAGTCCAATCG	
AaDuox2	CGTGGCGGAGTATTTCAGT	GCAAATTCATCAGCAACCAC	
**The primers for double-strand RNA synthesis**	**Upper primer**	**Lower primer**	
AaHig	TAATACGACTCACTATAGGGCTCACGACACGCCGTTCTG	TAATACGACTCACTATAGGGTCTCCTCATCTGGACCTTCGT	
GFP	TAATACGACTCACTATAGGGGTGAGCAAGGGCGAGGAG	TAATACGACTCACTATAGGGCATGATATAGACGTTGTGGCTGTT	
CpHig	TAATACGACTCACTATAGGGCAGCAAAAGTATCAATCGCA	TAATACGACTCACTATAGGGGTCAGCAGTGTAGTCGGAACG	
